# Kinetic asymmetry allows macromolecular catalysts to drive an information ratchet

**DOI:** 10.1038/s41467-019-11402-7

**Published:** 2019-08-23

**Authors:** R. Dean Astumian

**Affiliations:** Department of Physics, University of Maine, Orono, ME 04469-5709 USA

**Keywords:** Interlocked molecules, Self-assembly, Molecular machines and motors, Physical chemistry

## Abstract

Molecular machines carry out their function by equilibrium mechanical motions in environments that are far from thermodynamic equilibrium. The mechanically equilibrated character of the trajectories of the macromolecule has allowed development of a powerful theoretical description, reminiscent of Onsager’s trajectory thermodynamics, that is based on the principle of microscopic reversibility. Unlike the situation at thermodynamic equilibrium, kinetic parameters play a dominant role in determining steady-state concentrations away from thermodynamic equilibrium, and kinetic asymmetry provides a mechanism by which chemical free-energy released by catalysis can drive directed motion, molecular adaptation, and self-assembly. Several examples drawn from the recent literature, including a catenane-based chemically driven molecular rotor and a synthetic molecular assembler or pump, are discussed.

## Abstract (web summary)

The mechanism by which macromolecular catalysts use energy from exergonic reactions to move, adapt, and assemble has been unclear. In this Perspective article, R. Dean Astumian shows that in addition to disequilibrium of the catalyzed reaction, kinetic asymmetry is the essential feature required to drive non-equilibrium response by an information ratchet mechanism.

## Introduction

Living systems require continual input of energy. Some of this energy is used to create and to maintain structure and function, and the rest is wasted (dissipated). In recognition of the fact that all non-equilibrium processes dissipate energy, Prigogine coined the term “dissipative structures” to describe systems that use energy flow to maintain ordering and behavior that would not persist if the system were allowed to relax to equilibrium^1^. Examples of phenomena relevant for macromolecules that occur in the presence of energy flow, and by which order arises, include directed motion^2^, molecular adaptation^3^, and assembly of high free-energy structures^4^. There has been great progress recently in understanding how energy is absorbed and harnessed by molecular systems brought about by remarkable achievements in the design and synthesis of molecules that operate away from chemical equilibrium^5^. It is becoming evident that thermodynamically non-equilibrium phenomena can be best understood in the context of microscopic reversibility^6^.

Microscopic reversibility is a principle first enunciated by Tolman^7^ in the 1920’s, and highlighted by Onsager in his development of the reciprocal relations^8^ and in subsequent work with his student, Stefan Machlup, on trajectory-based thermodynamics^9^. Microscopic reversibility is general, arising because of the time reversibility of fundamental equations of motion—Newton’s or Schrodinger’s—but is especially useful under conditions where acceleration is negligible (low Reynold’s number regime^10^, i.e., mechanical equilibrium) and where velocities are equilibrated rapidly by thermal noise (thermal equilibrium^11^). These conditions, summarized by Onsager and Machlup as “The essential physical assumption about the irreversible processes is that they are linear; i.e., that the fluxes depend linearly on the forces that cause them”^9^, are ubiquitous in the solution phase molecular science on which this article is focused. It is important to understand Onsager and Machlup’s condition carefully^11,12^. There is no requirement that the averages of the fluxes depend linearly on the externally applied forces. Few systems would obey this criterion for even moderate applied forces. Instead, from the single-molecule perspective, the requirement is that the local fluxes (i.e., the velocities of some parts of the molecule relative to other parts) be linearly proportional to the local forces (the gradients of the molecular free-energy landscape^13^) that cause them. This condition is met if the motion of a single molecule is over-damped and governed by a single potential (an energy landscape), a condition fulfilled by essentially all chemically, but not optically^14^, driven molecular systems in solution. For a better understanding of these terms see Box 1.

A major point of the present perspective article can be summarized succinctly: at thermodynamic equilibrium the distribution among states of a system is determined solely by the free energies of the states and there are no net fluxes between the states; away from thermodynamic equilibrium the deviation of the distribution from the values that would pertain at equilibrium, as well as the fluxes between the states, is determined by the kinetic asymmetry of the system, i.e., by the relative heights of energy barriers between the states, as well as by the strength of the non-equilibrium driving^15,16^. As the environment of a macromolecule is driven far from equilibrium the macromolecule tends to occupy states of greatest kinetic, not thermodynamic, stability, and the adaptation to non-equilibrium conditions is independent of the free-energies of the states themselves.

## Information ratchets, kinetic stability, and kinetic asymmetry

**Fig. 1:**
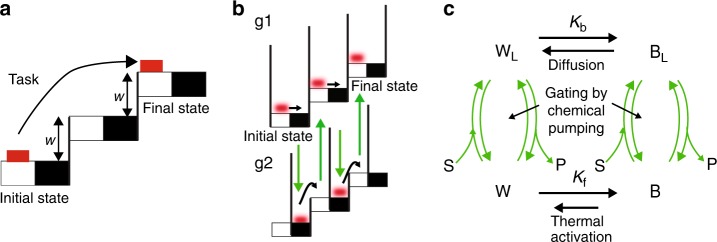
Comparison between a macroscopic task and a microscopic information ratchet. Comparison of **a** moving a brick up a staircase in the macroscopic world, in which losses through friction are inevitable, and **b** an information ratchet appropriate for description of a molecular machine in the microscopic world where diffusion provides a mechanism for motion without loss owing to friction but in which the second law forbids net directed motion without the input of energy, and **c** how this can be described in terms of the four state mechanism for a minimal Brownian machine. Each clockwise cycle through the four states describes the movement of the “brick” one step to the right, where W = white, B = black, S = substrate, and P = product, and subscript L denotes the “bound” form of the motor as S and P lose their individual identity once bound to the motor, and ${K}_{\mathrm{b}}\,{\mathrm{and}}\,{K}_{\mathrm{f}}$ are equilibrium constants between the black and white stairs, respectively. Directionality of cycling in **c** is controlled by the position dependence of the rates for the binding/dissociation processes of S and P

Three phrases occur time and time again in this perspective article—information ratchet, kinetic stability, and kinetic asymmetry. Let us clarify these concepts by comparing accomplishment of a macroscopic task with accomplishing a similar task in the microscopic world. Consider Fig. 1a in which the task of moving a brick from a low step to a high step is imagined. In the macroscopic world the brick could be pushed from the white to black section of the lowest step. This process would dissipate energy owing to friction between the brick and step. The brick could then be lifted to the next step, an energy input that, in principle, could be accomplished reversibly. Following elevation to the next step the brick would be pushed from the white to black section on the second step, once again dissipating energy because of friction, and then reversibly lifted to the third step to complete the task. The intrinsically irreversible steps are those in which the brick is pushed on the flat parts of the steps against the friction between the brick and step. To minimize dissipation (maximize efficiency) we would have to minimize this friction.

A similar task in the microscopic world can be accomplished in a very different way that utilizes thermal noise and diffusion (Fig. 1b) and requires only the manipulation of gates^17,18^. In this example a microscopic brick, positionally blurred because of diffusion, starts on the white section of the bottom stair with the gates in configuration g1. Diffusion owing to thermal noise allows the brick to move to the black section of the bottom step, where some feedback triggers the configuration of the gates to shift from g1 to g2. In configuration 2 the brick is prevented by the gate from moving back to the white area to the left. Because of thermal noise, the brick will eventually undergo thermal activation to the white area on the higher step to the right. Feedback owing to the presence of the brick on a white area triggers transition of the gates from g2 to g1, which prevents backward motion to the lower step to the left. Diffusion allows motion to the right, to the black area of the second brick, where once again feedback triggers a change in the configuration of the gates from g1 to g2 to prevent backward motion. Finally, thermal activation eventually brings the brick to the white region of the uppermost stair, triggering the switching of the gates from g2 to g1. This completes the task even though nothing describable as force times distance work has been done on the brick and there has been no dissipative loss owing to friction. There is neither pushing nor pulling involved, and describing the mechanism as “generating force” is clearly not appropriate even though the mechanism does allow input energy to do work against the applied load *w* The key feature is known as the “Brownian motor principle” according to which chemical energy is used to prevent backward motion rather than to cause forward motion^19^.

Two essential ingredients of this mechanism are diffusion and thermal activation, both of which occur because of thermal noise. The coefficient of viscous friction *γ* controls the timescale of these processes since diffusion ($D \propto \gamma ^{ - 1}$, fluctuation dissipation theorem) and thermal activation ($k_{{\mathrm{act}}} \propto \gamma ^{ - 1}e^{ - {{\Delta }}\mathrm{U}}$, Kramer’s formula^12^) are both related to the coefficient of viscous friction, where here and elsewhere in this paper energies are given in units of the thermal energy, *k*_B_*T*. Nevertheless, there is no energy loss owing to friction—the physical motions of the brick, both diffusive and thermally activated, are mechanical equilibrium processes.

The final state is kinetically more stable than the initial state despite the fact that the initial state is thermodynamically more stable owing to the lower potential energy of the brick. This kinetic stabilization arises through correlations between the position of the brick and the configuration of the gates, hence the description “information ratchet”^17^. Leigh and colleagues^20^ have synthetically implemented a light-driven information ratchet, and subsequently chemically driven molecular information ratchets^21,22^ have been described, all based on mechanically bonded molecules^23^.

It is of course possible to use, e.g., lasers^24^ to detect the location of a microscopic particle and to enforce gating, but in the original proposal for an information ratchet^17,18^, and of the most relevance for molecular systems, the specific mechanism for controlling the gates is allosteric interaction between the particle and the track. A kinetic model for a minimal Brownian motor^25,26^ describes this information ratchet as shown in Fig. 1c, where the steps involving diffusion, thermal activation, and chemical pumping to control the gates are explicitly shown. The transitions between the “bound” and “free” states, written with curved arrows in Fig. 1c, involve catalysis of a substrate S to a product P

$${\mathrm{S}} + {\mathrm{W}}\mathop { \rightleftharpoons }\limits^{K_{{\mathrm{W,S}}}} {\mathrm{W}}_{\mathrm{L}}\mathop { \rightleftharpoons }\limits^{K_{{\mathrm{W,P}}}^{ - 1}} {\mathrm{W}} + {\mathrm{P}}\,\,\,\,{\mathrm{ and }}\,\,\,\,{\mathrm{S}} + {\mathrm{B}}\mathop { \rightleftharpoons }\limits^{K_{{\mathrm{B,S}}}} {\mathrm{B}}_{\mathrm{L}}\mathop { \rightleftharpoons }\limits^{K_{{\mathrm{B,P}}}^{ - 1}} {\mathrm{B}} + {\mathrm{P}}$$

where $K_{{\mathrm{W,S}}}K_{{\mathrm{W,P}}}^{ - 1} = K_{{\mathrm{B,S}}}K_{{\mathrm{B,P}}}^{ - 1} = K_{{\mathrm{eq}}}$. The ratios of forward to backward rate constants, i.e., the equilibrium constants, are set by thermodynamics and can be written $K_{{\mathrm{W,S}}} = \frac{{k_{{\mathrm{W,}} + 1}}}{{k_{{\mathrm{W,}} - 1}}},K_{{\mathrm{W,P}}} = \frac{{k_{{\mathrm{W,}} - 2}}}{{k_{{\mathrm{W,}} + 2}}},K_{{\mathrm{B,S}}} = \frac{{k_{{\mathrm{B,}} + 1}}}{{k_{{\mathrm{B,}} - 1}}},K_{{\mathrm{B,P}}} = \frac{{k_{{\mathrm{B,}} - 2}}}{{k_{{\mathrm{B,}} + 2}}}$, where the cycle conditions $K_{{\mathrm{W,S}}}K_{\mathrm{b}}K_{{\mathrm{B,S}}}^{ - 1}K_{\mathrm{f}}^{ - 1} = K_{{\mathrm{W,P}}}K_{\mathrm{b}}K_{{\mathrm{B,P}}}^{ - 1}K_{\mathrm{f}}^{ - 1} = 1$ are necessary to insure thermodynamic consistency. The absolute magnitudes of the rate constants, however, and hence the kinetic asymmetry can be controlled by evolution for biomolecular machines or by chemical design in the case of synthetic molecular machines. The kinetic asymmetry can be expressed in terms of the ratios of the “off” rates $\frac{{k_{{\mathrm{W,}} + 2}}}{{k_{{\mathrm{W,}} - 1}}}$ and $\frac{{k_{{\mathrm{B,}} + 2}}}{{k_{{\mathrm{B,}} - 1}}}$. These ratios are independent of the concentrations [S] and [P] and of the equilibrium constants. If $\frac{{k_{{\mathrm{W,}} + 2}}}{{k_{{\mathrm{W,}} - 1}}} \ll 1$ and $\frac{{k_{{\mathrm{B,}} + 2}}}{{k_{{\mathrm{B,}} - 1}}} \gg 1$ we picture a situation in which substrate binds (and dissociates) quickly when the brick is on a white square but generally waits until it is on a black square to dissociate product. This position dependence of the rates leads to clockwise cycling in Fig. 1c, i.e., left to right uphill motion of the brick in Fig. 1b. Such position dependence can arise from allosteric interactions and describes the kinetic asymmetry required for a macromolecular catalyst to act as an energy transducer to allow chemical pumping. There are four clockwise trajectories starting and ending at the same state and involving all four states—S binds, P is released; P binds, S is released; S binds, S is released; and P binds, and P is released. Each of these has a microscopic reverse counter-clockwise cycle. The challenge then is to calculate the average likelihood of a clockwise to counter-clockwise cycle using the principle of microscopic reversibility. In order to see how this plays out quantitatively let us first focus on kinetic asymmetry of a single Michaelis–Menten (MM) enzyme.

## Kinetic asymmetry of a MM enzyme and design of information ratchets

**Fig. 2:**
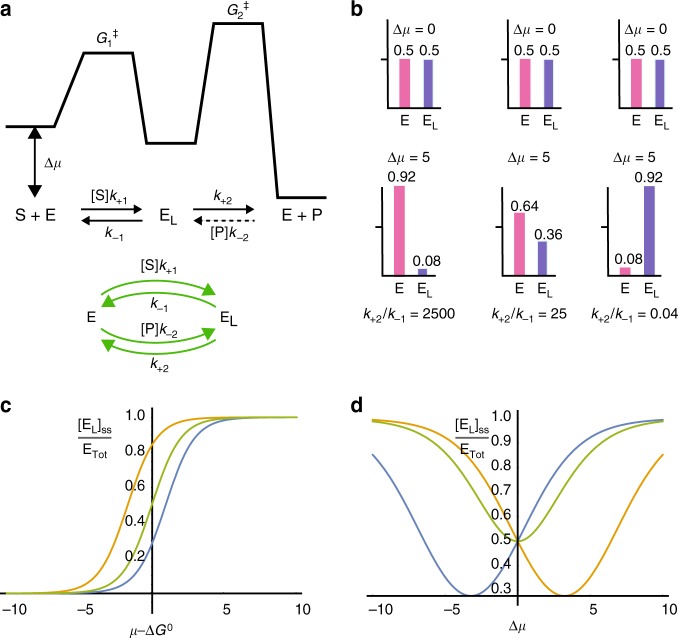
Kinetic asymmetry and Michaelis–Menten enzymes. **a** Energy profile for a Michaelis–Menten mechanism for catalysis, and two equivalent ways of writing the mechanism. An important quantity for determining the non-equilibrium behavior of the enzyme is the difference in transition state free-energies, $\Delta G^\ddagger = G_1^\ddagger - G_2^\ddagger$, which can be expressed in terms of the rate constants as $\frac{{k_{ - 1}}}{{k_{ + 2}}} = {\boldsymbol{e}}^{ - \Delta G^\ddagger }$. This ratio does not depend on either *μ* or Δ*μ*. **b** Bar graphs illustrating the equilibrium ($\Delta {\mu } = 0$) distribution, where the kinetic asymmetry ${k}_{ + 2}/{k}_{ - 1}$ has no role, and the non-equilibrium ($\Delta {\mu } = 5$) steady-state distributions that strongly depend on the kinetic asymmetry ${k}_{ + 2}/{k}_{ - 1}$. **c**, **d** The concentrations of substrate (S) and product (P) are taken as constant (i.e., chemo-stated) in all calculations, and can be written in terms of the chemical potential using activity coefficients $[{\mathrm{S}}] = {a}_{\mathrm{S}}{e}^{{\mu } + {\Delta \mu }}$ and $[{\mathrm{P}}] = {a}_{\mathrm{P}}{e}^{{\mu } - {\Delta \mu }}$. In ideal solutions both activity coefficients are ~ 1 in the units of concentration in which [S] and [P] are specified. Two types of enzyme adaptation: **c** “equilibrium” adaptation where the binding is based on the reference chemical potential ${\mu } - \Delta {G}^{0},\Delta {G}^0 = ({G}_{{\mathrm{E}}_{\mathrm{L}}}^0-{G}_{\mathrm{E}}^0)$, with ${k}_{ - 1} = 5$, ${k}_{ + 2} = 0.2$, and $\Delta {{\mu }} = 3.4$ (orange); $\Delta {\mu } = 0$ (green); and $\Delta {\mu } = - 3.4$ (blue). **d** Non-equilibrium adaptation governed by Δ*μ* plotted at fixed ${\mu } - \Delta G^0 = 0$. The binding is controlled by $\Delta {\mu }$ and by the kinetic asymmetry (${k}_{ + 2}/{k}_{ - 1} = 25\,({\mathrm{blue}}); \,{k}_{ + 2}/{k}_{ - 1} = 1\,({\mathrm{green}}); \,{\mathrm{and}}\,{k}_{ + 2}/{k}_{ - 1} = 0.04\,({\mathrm{orange}})$)

Consider the MM mechanism^25,27^ ${\mathrm{S}} + {\mathrm{E}}\mathop { \rightleftharpoons }\limits^{K_{\mathrm{S}}} {\mathrm{E}}_{\mathrm{L}}\mathop { \rightleftharpoons }\limits^{K_{\mathrm{P}}^{ - 1}} {\mathrm{E}} + {\mathrm{P}}$ for enzyme catalysis, where $K_{\mathrm{S}}K_{\mathrm{P}}^{ - 1} = K_{{\mathrm{eq}}}$, illustrated in Fig. 2a. This MM mechanism describes a macromolecule that binds substrate, S, or product, P to form a Michaelis complex ${\mathrm{E}}_{\mathrm{L}}$. The enzyme facilitates inter-conversion between substrate and product by providing a path with a lower energy barrier than that for the uncatalyzed process. The Michaelis complex is represented as ${\mathrm{E}}_{\mathrm{L}}$ rather than the more traditional ${\mathrm{E}}_{\mathrm{S}}$ to reflect the fact that ligand loses its individual identity as substrate or product when bound to the enzyme^28^. At steady state the ratio between the concentrations is

1 $$\frac{{\left[ {{\mathrm{E}}_{\mathrm{L}}} \right]|_{{\mathrm{ss}}}}}{{\left[ {\mathrm{E}} \right]|_{{\mathrm{ss}}}}} = \frac{{\left( {k_{ + 1}\left[ {\mathrm{S}} \right] + k_{ - 2}\left[ {\mathrm{P}} \right]} \right)}}{{\left( {k_{ - 1} + k_{ + 2}} \right)}},$$

where the two pathways from ${\mathrm{E}}$ to ${\mathrm{E}}_{\mathrm{L}}$ are binding S and binding P. We define equilibrium constants for both S and P binding, $\frac{{k_{ + 1}}}{{k_{ - 1}}} = K_{\mathrm{S}},\frac{{k_{ - 2}}}{{k_{ + 2}}} = K_{\mathrm{P}}$, which, together with the thermodynamic constraint $\frac{{[{\mathrm{S}}]}}{{[{\mathrm{P}}]}}\,\frac{{K_{\mathrm{S}}}}{{K_{\mathrm{P}}}} = e^{{{\Delta }}\mu }$, can be used to rewrite the steady-state ratio

2 $$\frac{{\left. {[{\mathrm{E}}_{\mathrm{L}}]} \right|_{{\mathrm{ss}}}}}{{\left. {[{\mathrm{E}}]} \right|_{{\mathrm{ss}}}}} = [{\mathrm{S}}]K_{\mathrm{S}}\left[ {\frac{{\left( {\frac{{{{k}}_{ - 1}}}{{{{k}}_{ + 2}}} + e^{ - {{\Delta }}\mu }} \right)}}{{\left( {\frac{{{{k}}_{ - 1}}}{{{{k}}_{ + 2}}} + 1} \right)}}} \right].$$

The term in brackets has such an important role for understanding how catalysis drives non-equilibrium function such as directed motion, assembly, and adaptation, that we assign it a specific name and symbol, the kinetic asymmetry parameter,

3 $${\cal{A}} = \frac{{\left( {\frac{{k_{ - 1}}}{{k_{ + 2}}} + e^{ - \Delta {\mathrm{\mu }}}} \right)}}{{\left( {\frac{{k_{ - 1}}}{{k_{ + 2}}} + 1} \right)}}$$

Kinetic asymmetry is key to understanding directionality of kinetic cycles under non-equilibrium conditions, and pertains to all catalytic mechanisms, not only the MM mechanism for enzymes chosen here for its ubiquity and simplicity. Note that ${\cal{A}}$ depends only on Δ*μ* and on the ratio of “off ” rates $\frac{{k_{ - 1}}}{{k_{ + 2}}}$, and is independent of the free energies of the states themselves. In thermodynamic equilibrium, kinetic asymmetry is irrelevant as shown at the top of Fig. 2b. In sharp contrast, with $\Delta \mu \ne 0$ the ratio $\frac{{k_{ - 1}}}{{k_{ + 2}}}$ determines the steady-state levels as shown at the bottom of Fig. 2b. For the standard Michaelis–Menten reaction mechanism $${\mathrm{S}} + {\mathrm{E}}\begin{array}{*{20}{c}} {[{\mathrm{S}}]k_{ + 1}} \\ \rightleftharpoons \\ {k_{ - 1}} \end{array}{\mathrm{E}}_{\mathrm{L}}\begin{array}{*{20}{c}} {k_{ + 2}} \\ \rightarrow \\ {} \end{array}{\mathrm{E}} + {\mathrm{P}}$$ written for initial kinetics (i.e., with [P] $\to$ 0) the steady-state catalytic velocity $v_{{\mathrm{cat}}} = \frac{{k_{ + 2}{\mathrm{E}}_{{\mathrm{Tot}}}[{\mathrm{S}}]}}{{1/\left( {K_{\mathrm{S}}{\cal{A}}} \right) + [{\mathrm{S}}]}}$ is the standard expression with $K_{\mathrm{M}} = 1/\left( {K_{\mathrm{S}}{\cal{A}}} \right)$.

The concentration of bound enzyme is determined by the levels of substrate and product in two distinct ways. First, at fixed Δ*μ*, the concentration of bound enzyme is a sigmoidal function of the reference chemical potential as shown in Fig. 2c. With Δ*μ* = 0 (green curve in 2c) the inflection point is at $\mu = \Delta G^0$, but at different values of Δ*μ* the inflection point can be at lower or higher values of *μ* depending on the kinetic asymmetry of the system and on the value of Δ*μ*. This sigmoidal dependence reflects equilibrium adaptation even when $\Delta \mu \ne 0$. At fixed $\mu$, Δ*μ* influences the concentration $\left. {[{\mathrm{E}}_{\mathrm{L}}]} \right|_{{\mathrm{ss}}}$ non-monotonically (Fig. 2d), in a way that is controlled by kinetics (the ratio of “off” rate constants, $k_{ + 2}/k_{ - 1} = e^{G_1^\ddagger - G_2^\ddagger }$) rather than by thermodynamics. The change in the ratio of bound to free states of the enzyme as a function of Δ*μ* and of the kinetic asymmetry reflects non-equilibrium adaptation and provides a mechanism by which free energy released by conversion of a substrate to product can be utilized to drive molecular machines^17,25^. Returning to the example in Fig. 1c we recognize that the kinetic asymmetry parameters are ${\cal{A}}_{\mathrm{W}} = \left[ {\frac{{\left( {\frac{{k_{{\mathrm{W, - 1}}}}}{{k_{{\mathrm{W, + 2}}}}} + e^{ - \Delta \mu }} \right)}}{{\left( {\frac{{k_{{\mathrm{W,}} - 1}}}{{k_{{\mathrm{W,}} + 2}}} + 1} \right)}}} \right]$ and ${\cal{A}}_{\mathrm{B}} = \left[ {\frac{{\left( {\frac{{k_{{\mathrm{B,}} - 1}}}{{k_{{\mathrm{B,}} + 2}}} + e^{ - \Delta \mu }} \right)}}{{\left( {\frac{{k_{{\mathrm{B,}} - 1}}}{{k_{{\mathrm{B,}} + 2}}} + 1} \right)}}} \right]$. In addition to the product of the four equilibrium constants, which must be unity, the ratio of probabilities for a clockwise and counter-clockwise cycle picks up a factor $\frac{{{\cal{A}}_{\mathrm{W}}}}{{{\cal{A}}_{\mathrm{B}}}}$, which is different than unity only when $\Delta \mu \ne 0$, and for $\Delta \mu > 0$ can be greater than, less than, or equal one depending on whether $\frac{{k_{{\mathrm{W, - 1}}}}}{{k_{{\mathrm{W, + 2}}}}}\frac{{k_{{\mathrm{B,}} + 2}}}{{k_{{\mathrm{B,}} - 1}}}$ is greater than, less than, or equal unity. We can better recognize the importance of this factor by considering an experimental example of a catalysis-driven synthetic rotor.

Box 1 Glossary **Adaptation -** Adjustment of a system to changing conditions. Molecular switching is an example of equilibrium adaptation. Non-equilibrium adaptation can be more complex, with even non-monotonic dependence on the "distance from equilibrium".

**Catenanes and rotaxanes** - A catenane is a molecule in which two or more rings are mechanically bonded to (interlinked with) one another, and a rotaxane is a dumbbell-shaped molecule that is threaded through one or more macrocycle rings with stopper groups covalently attached to the two ends of the rod to prevent the rings from escaping.

**Detailed balance** - Condition according to which the probability for a forward transition is equal the probability for a reverse transition, $k_{\rm{il}}[{\rm{i}}] = k_{\rm{li}}[{\rm{l}}]$. When applied to individual cycles in a kinetic network detailed balance holds if the cycle affinity is zero irrespective of whether the overall system is in equilibrium.

**Local detailed balance** - Assertion that ratios of effective forward and backward rate constants are proportional to the exponential of a free-energy driving term such as a chemical potential difference of substrate and product. This condition is not generally valid.

**Directed motion -** Continual motion or rotation in the same direction. Persistent directional motion is impossible without a source of free energy and is hence intrinsically a thermodynamically non-equilibrium phenomenon.

**Dissipation** - Process in which free-energy is wasted, becoming irrecoverable, and unavailable for carrying out useful processes.

**Energy ratchet** - Mechanism in which a spatially asymmetric energy landscape is modulated in time to produce directed motion.

**Free-energy** - Energy that is available to carry out useful processes such as directed motion, self-assembly, and non-equilibrium adaptation.

**Information ratchet** - Mechanism by which directionality is achieved by controlling energy barriers, either by raising or lowering the barrier depending on the state of the system, or by engineering the energy landscape to kinetically favor a specific pathway through the states and then provide chemical energy to allow directional motion on that pathway by mass action.

**Mass action -** Idea that for catalysis-driven processes, the directional rate along the preferred path is proportional to the concentration of substrate of the catalyzed reaction.

**Mechanical bond** - Physical interlocking of components of a molecule such that they cannot escape from one another even without any specific chemical interaction between the components.

**Mechanical equilibrium** - Occurs when there is no acceleration. Motion at low Reynold's number is at mechanical equilibrium since the viscous force is much greater than the inertial force (mass times acceleration). Mechanical equilibrium holds for almost all molecular processes in solution.

**Microscopic reversibility** - Principle that arises from the fundamental time symmetry of the underlying equations of motion describing the dynamics of a system, either Newton's equation or Schrodinger's equation.

**Power stroke -** Visco-elastic conformational change incorrectly thought to be important for directional motion of many ATP hydrolysis driven molecular machines. An external energy source lifts the macromolecular machine into a high-energy state from which it undergoes directional relaxation thereby "producing torque or force". Light-driven motors generally do operate by a power-stroke mechanism.

**Self-assembly -** Assembly of a structure without external manipulation that is, in and of itself, more ordered than the precursors from which it is constructed. In spontaneous assembly disordering of other components of the overall system (e.g., water) to make the process one involving overall release of free-energy and hence to proceed, but fuel-driven assembly requires external energy from time dependent modulation or catalysis of an exergonic process.

**State function** - A function describing a transition (e.g., basic free-energy change) that depends only on the initial and final states of the system and not on the specific trajectory by which the transition occurs.

**Trajectory** - Sequence of transitions between states of a molecule. A cyclic trajectory starts and ends in the same state.

**Thermodynamic consistency** - kinetic and thermodynamic parameters must reflect the cycle condition, e.g., for ${\rm{ j \to i \to l \to k \to j}},\Delta G_{\rm{ji}} + \Delta G_{\rm{il}} + \Delta G_{\rm{lk}} + \Delta G_{\rm{kj}} = 0$ or equivalently $K_{\rm{ji,S}}K_{\rm{il}}K_{\rm{lk,S}}K_{kj} = K_{\rm{ji,P}}K_{\rm{il}}K_{\rm{lk,P}}K_{\rm{kj}} = 1$. Away from eq. $K_{\rm{ji,S}}K_{\rm{il}}K_{\rm{lk,P}}K_{\rm{kj}} \ne 1.$

**Thermal equilibrium** - Occurs when the total kinetic energy of a system is, on average, equally distributed among all degrees of freedom such that the system obeys the equipartition theorem. Thermal equilibrium is ubiquitous and almost always holds.

**Thermodynamic equilibrium** - Occurs when a system is distributed among its states according to a Boltzmann distribution, and where the chemical potential of all molecules is the same as one another. Thermodynamic equilibrium is a dynamic state in which all possible transitions occur but where each process is exactly as likely as its microscopic reverse. Most biological systems are not in thermodynamic equilibrium.

## Catalysis-driven molecular rotor

**Fig. 3:**
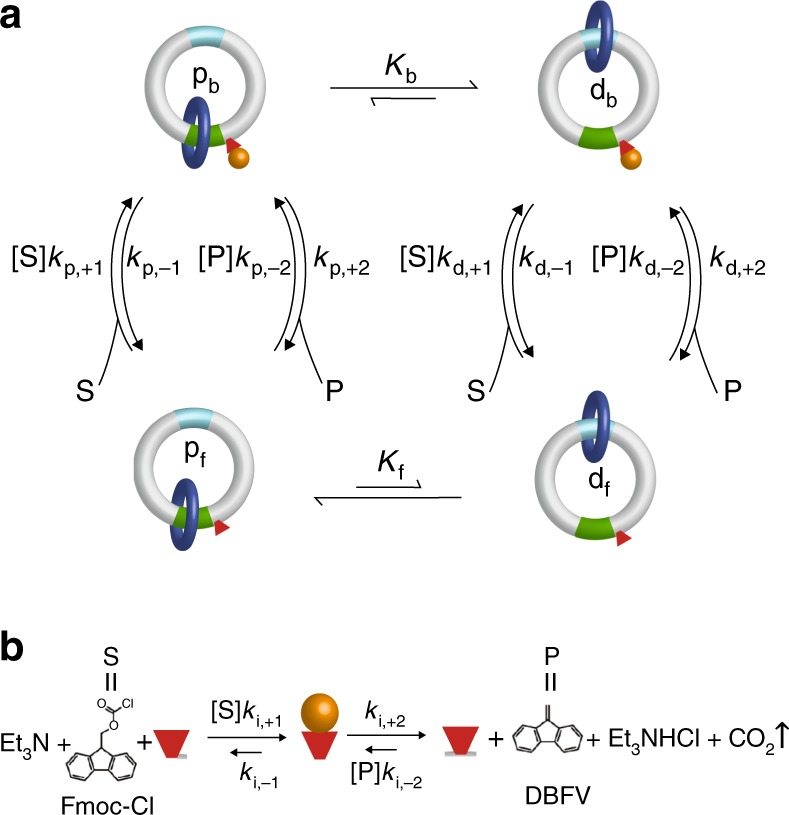
Four state synthetic rotor driven by a catalyzed reaction. **a** The rotor is based on that of Wilson et al.^29^ where a small benzylic amide macrocycle, shown as a dark blue ring, undergoes switching transitions between two different recognition sites, one shown as green and the other aqua. A catalytically active moiety near the green recognition site facilitates conversion of Fmoc-Cl to DBFV by a reaction shown in **b**. The symbol “p” indicates proximal, “d” indicates distal, “b” indicates that the catalytic site is bound, and “f” indicates that the catalytic site is free. The chemical potential difference driving conversion of Fmoc-Cl to DBFV under the experimental conditions of Wilson et al.^28^ is $\Delta {\mu } \approx 25$, slightly greater than the chemical potential difference driving ATP hydrolysis under physiological conditions, so the reaction is “far from equilibrium”. A key question is whether the blue ring undergoes clockwise or counter-clockwise rotation and what properties determine the direction of rotation?

Consider the catalysis-driven rotor, based on the rotor described by Wilson et al.^29^, shown in Fig. 3a. This rotor is a minimal Brownian machine^25^ where the two-state catalytic transition [bound (b) ⇔ free (f)] that enforces kinetic gating—the ring cannot pass over the occupied catalytic site—is coupled to a two-state mechanical switch [proximal (p) $\rightleftharpoons$ distal (d)]. The most obvious question one can ask about such a rotor is what direction, if any, does the dark blue ring rotate when the rotor is provided with its chemical fuel 9-Fluoroenylmethoxycarbonyl Chloride (Fmoc). The driving reaction is shown in Fig. 3b, where Fmoc is substrate S, Dibenzofulvene is product P, and the bound form is represented by an orange sphere. The distinct recognition sites, aqua and green, for the dark blue ring, along with the catalytic active site, shown as a red cone, provide a reference for answering this question based on the order in which the dark blue ring visits the sites, aqua → red → green → aqua (clockwise), versus aqua → green → red → aqua (counter-clockwise). The ratio of the net probabilities for clockwise (${\mathrm{p}}_{\mathrm{f}} \to {\mathrm{p}}_{\mathrm{b}} \to {\mathrm{d}}_{\mathrm{b}} \to {\mathrm{d}}_{\mathrm{f}} \to {\mathrm{p}}_{\mathrm{f}}$) to counter-clockwise (${{\mathrm{p}}_{\mathrm{f}} \to {\mathrm{d}}_{\mathrm{f}} \to {\mathrm{d}}_{\mathrm{b}} \to {\mathrm{p}}_{\mathrm{b}} \to {\mathrm{p}}_{\mathrm{f}}}$) cycles is given by the ratio of the products of the net transition constants

4 $$r_0 = K_{\mathrm{b}}K_{\mathrm{f}}^{ - 1}\frac{{\left( {[{\mathrm{S}}]k_{{\mathrm{p,}} + 1} + [{\mathrm{P}}]k_{{\mathrm{p,}} - 2}} \right)}}{{\left( {k_{{\mathrm{p,}} - 1} + k_{{\mathrm{p,}} + 2}} \right)}}\,\frac{{\left( {k_{{\mathrm{d,}} + 2} + k_{{\mathrm{d,}} - 1}} \right)}}{{\left( {\left[ {\mathrm{S}} \right]k_{{\mathrm{d,}} + 1} + [{\mathrm{P}}]k_{{\mathrm{d,}} - 2}} \right)}}$$

The quantity *r*_0_ is known as the intrinsic directionality^16^, or the ratcheting constant^4^, and is the key parameter for evaluating the effectiveness of energy transduction. If $r_0 > 1$ the net cycling is in the clockwise direction and if $r_0 < 1$ the net cycling is in the counter-clockwise direction. If the rotation is configured to perform work, *w*, on the environment as is the case with many biomolecular machines, the net directionality is $r = r_0e^{ \pm w}$ where we take “ + ” if $r_0 < 1$ and “−” if $r_0 > 1$ so that the net catalysis-driven directionality performs work in the environment. Examination of the rotor in Fig. 3 reveals two obvious design features—the thermodynamic interaction between the ring and the Fmoc, and a kinetic effect owing to the steric influence of the ring when on the proximal site.

### Plausible but wrong hypothesis for catalysis-driven cycling

From a thermodynamic perspective, the green recognition site is selected to be a priori more stable than the aqua site such that $K_{\mathrm{f}} \, < \, 1$, but a repulsive interaction between the dark blue ring and the orange sphere (bound intermediate of the conversion between Fmoc-Cl and DBFV) reverses the stability so that $K_{\mathrm{b}} > 1$. This arrangement of equilibrium constants seems to predict that the rotation should be clockwise. It is easy to come up with a “just-so story” in which the chemical energy released by catalytic conversion of Fmoc-Cl to DBFV forces the binding of Fmoc-Cl to the initial state p_f_ despite the repulsion, thus converting the molecule to a high free-energy intermediate p_b_ from which the system relaxes to state d_b_ by clockwise mechanical rotation of the blue ring reasonably termed a power stroke. After dissociation of DBFV, the molecule is in the state d_f_, but the blue ring can then relax to the a priori most stable green site, thereby resetting the system to the state p_f_ to continue this clockwise rotation so long as Fmoc-Cl is in excess. This verbal description is consistent with quantitative analysis based on assigning the rate constants $[{\mathrm{S}}]k_{{\mathrm{p,}} + 1} = k_{{\mathrm{p,}} + 2} = [{\mathrm{S}}]k_{{\mathrm{d,}} + 1} = k_{{\mathrm{d,}} + 2} = e^{ + \frac{{\Delta \mu }}{4}}\,{\mathrm{and}}\,[{\mathrm{P}}]k_{{\mathrm{p,}} - 2} = k_{{\mathrm{p,}} - 1} = [{\mathrm{P}}]k_{{\mathrm{d,}} - 2} = k_{{\mathrm{d,}} - 1} = e^{ - \frac{{\Delta \mu }}{4}}$. Inserting these expressions into Eq. (4) we find $r > 1$, and in the limit of large Δ*μ*, $r\mathop {{\lim }}\limits_{\Delta {\mathrm{\mu }} \to \infty } \to K_{\mathrm{b}}K_{\mathrm{f}}^{{\mathrm{ - 1}}}$, consistent with clockwise cycling owing to Fmoc “pushing” the mobile ring when Fmoc binds.

### Correct theory

In fact, the molecular rotor preferentially cycles in the counter-clockwise direction. The mechanism involves kinetic gating rather than “pushing” the ring by electrostatic interactions. As explained by Wilson et al.^29^, the gating by which the kinetic asymmetry arises is owing to steric interaction between the protecting group and the blue ring. This effect is subtler than the repulsion based “power-stroke” model for clockwise rotation, but it turns out in experiment to be the correct description of the catalysis-driven rotation. When the blue ring is at the green recognition site the binding (and release) of Fmoc-Cl is hindered in comparison with the same reaction with the amine next to the green site occurring when the blue ring is at the aqua site, i.e., $k_{{\mathrm{p}}, + 1} \, < \, k_{{\mathrm{d,}} + 1}$ and $k_{{\mathrm{p,}} - 1} < k_{{\mathrm{d,}} - 1}$. The binding and release of DBFV, however, occurs at a site several bonds removed from the green recognition site and hence these rates are less strongly influenced, i.e., $k_{{\mathrm{p,}} + 2} \approx k_{{\mathrm{d,}} + 2}$ and $k_{{\mathrm{p,}} - 2} \approx k_{{\mathrm{d,}} - 2}$. Thus, we have $\frac{{k_{{\mathrm{p,}} + 2}}}{{k_{{\mathrm{p,}} - 1}}} > \frac{{k_{{\mathrm{d,}} + 2}}}{{k_{{\mathrm{d,}} - 1}}}$. This arrangement of rate constants describes a molecular information ratchet and correctly predicts counter-clockwise rotation since binding Fmoc-Cl is fast when the blue ring is distal to the active site, but the dissociation rate for DBFV is roughly the same irrespective of whether the blue ring is distal or proximal to the catalytic site.

### Quantitative understanding

To better understand why rotation is controlled by kinetic asymmetry rather than by thermodynamic interactions that influence the free-energies of the states, let us roll up our sleeves and carry out a quantitative analysis of this rotor. The equilibrium constants, $\frac{{k_{{\mathrm{p,}} + 1}}}{{k_{{\mathrm{p,}} - 1}}} = K_{{\mathrm{p,S}}},\frac{{k_{{\mathrm{p,}} - 2}}}{{k_{{\mathrm{p,}} + 2}}} = K_{{\mathrm{p,P}}},\frac{{k_{{\mathrm{d,}} + 1}}}{{k_{{\mathrm{d,}} - 1}}} = K_{{\mathrm{d,S}}},\frac{{k_{{\mathrm{d,}} - 2}}}{{k_{{\mathrm{d,}} + 2}}} = K_{{\mathrm{d,P}}},{\mathrm{where}}\,\frac{{K_{{\mathrm{p,S}}}}}{{K_{{\mathrm{p,P}}}}} = \frac{{K_{{\mathrm{d,S}}}}}{{K_{{\mathrm{d,P}}}}} = K_{{\mathrm{eq}}},$ must obey the constraints

5 $$\frac{{[{\mathrm{S}}]}}{{[{\mathrm{P}}]}}\,\frac{{K_{{\mathrm{p,S}}}}}{{K_{{\mathrm{p,P}}}}} = \frac{{[{\mathrm{S}}]}}{{[{\mathrm{P}}]}}\frac{{K_{{\mathrm{d,S}}}}}{{K_{{\mathrm{d,P}}}}} = e^{{\mathrm{\Delta }}\mu },$$

6 $$\frac{{K_{\mathrm{b}}}}{{K_{\mathrm{f}}}}\frac{{K_{{\mathrm{p,S}}}}}{{K_{{\mathrm{d,S}}}}} = \frac{{K_{\mathrm{b}}}}{{K_{\mathrm{f}}}}\frac{{K_{{\mathrm{p,P}}}}}{{K_{{\mathrm{d,P}}}}} = 1.$$

These constraints assure that the mechanism with its associated rate constants is thermodynamically consistent^15,17^. Eq. (5) reflects the fact that a catalyst does not change the equilibrium constant of the catalyzed reaction, whereas Eq. (6) is obtained from the thermodynamic boxes formed by separately considering binding/dissociation of S or binding/dissociation of P in which there is no release of chemical energy. Using Eqs. (5) and (6) the intrinsic directionality can be rewritten^15,16^ as

7 $$r_0 = \underbrace {\left[ {\frac{{\left( {\frac{{k_{{\mathrm{d}}, - 1}}}{{k_{{\mathrm{d}}, + 2}}} + 1} \right)}}{{\left( {\frac{{k_{{\mathrm{d}}, - 1}}}{{k_{{\mathrm{d}}, + 2}}} + e^{ - \Delta \mu} } \right)}}} \right]}_{{\cal{A}}_{\mathrm{d}}^{ - 1}}\,\underbrace {\left[ {\frac{{\left( {\frac{{k_{{\mathrm{p}}, - 1}}}{{k_{{\mathrm{p}}, + 2}}} + e^{ - \Delta \mu} } \right)}}{{\left( {\frac{{k_{{\mathrm{p}}, - 1}}}{{k_{{\mathrm{p}}, + 2}}}+1} \right)}}} \right]}_{{\cal{A}}{\mathrm{p}}}$$

The parameter $r_0$—the ratio of the kinetic asymmetry factors for the two catalytic configurations, d and p, discussed in the context of the MM mechanism in the previous section—is entirely specified by ${\mathrm{\Delta }}\mu$ and by the ratios of the “off” rates $\left( {\frac{{k_{{\mathrm{d,}} - 1}}}{{k_{{\mathrm{d,}} + 2}}}} \right)\,{\mathrm{and}}\,\left( {\frac{{k_{{\mathrm{p,}} - 1}}}{{k_{{\mathrm{p,}} + 2}}}} \right)$, which are independent of concentrations of S and P. For ${\mathrm{\Delta }}\mu > 0,{\mathrm{if}}\,\left( {\frac{{k_{{\mathrm{d,}} - 1}}}{{k_{{\mathrm{d,}} + 2}}}} \right) < \left( {\frac{{k_{{\mathrm{p,}} - 1}}}{{k_{{\mathrm{p,}} + 2}}}} \right)\,{\mathrm{then}}\,r_0 > 1$, and if, as is the case in the experimental rotor^28^,$\left( {\frac{{k_{{\mathrm{d,}} - 1}}}{{k_{{\mathrm{d,}} + 2}}}} \right) > \left( {\frac{{k_{{\mathrm{p,}} - 1}}}{{k_{{\mathrm{p,}} + 2}}}} \right)\,{\mathrm{then}}\,r_0 < 1$. When $\frac{{k_{{\mathrm{d,}} - 1}}}{{k_{{\mathrm{d,}} + 2}}} = \frac{{k_{{\mathrm{p,}} - 1}}}{{k_{{\mathrm{p,}} + 2}}}$ the net currents between the states are zero, and the steady-state distribution is the same as the equilibrium distribution, irrespective of Δ*μ* and of possibly large dissipation owing to catalysis. Note that all catalysis-driven motors function as information ratchets, the directionality, maximum load, and thermodynamic efficiency of which are governed by kinetic asymmetry^30^.

How can the simple, common sense, analysis based on electrostatic repulsion between the blue ring and the protecting group, and seemingly supported by calculation of the directionality parameter $r_0$ with plausible rate constants based on the system being “far from equilibrium” be so wrong? The answer is that despite the large Δ*μ* driving the catalysis, both Eqs. (5) and (6) must hold. The assignment of the rate constants $[{\mathrm{S}}] k_{{\mathrm{p,}} + 1} = k _{{\mathrm{p,}} + 2} = [{\mathrm{S}}]k _{{\mathrm{d,}} + 1} = k _{{\mathrm{d,}} + 2} = e^{ + \frac{{\Delta \mu }}{4}}\,{\mathrm{and}}\,[{\mathrm{P}}]k\, _{{\mathrm{p,}} - 2} = k\,_{{\mathrm{p,}} - 1} = [{\mathrm{P}}] k_{{\mathrm{d,}} - 2} = k _{{\mathrm{d,}} - 1} = e^{ - \frac{{\Delta \mu }}{4}}$ is indeed consistent with Eq. (5), but not with Eq. (6). If the protecting moiety Fmoc repels the blue ring such that $K_{\mathrm{b}} > \, K_{\mathrm{f}}$, then the dissociation constants must also reflect this repulsion, $K_{{\mathrm{d,S}}} \, > \, K_{{\mathrm{p,S}}}$ and $K_{{\mathrm{d,P}}} \, > \, K_{{\mathrm{p,P}}}$, in such a way that Eq. (6) is obeyed. We also recognize that another very commonly asserted model known as “local detailed balance”^31^ is wrong as discussed below. These seeming minutiae regarding quantitative analysis of models for molecular machines, or at least the conclusions arising from them, are very important for synthetic chemists because they reveal that the essential design feature for catalytically driven molecular motors is kinetic asymmetry owing to allosteric interaction, and not the incorporation of a power stroke^30,32^, an insight that was key to the design of the rotor of Wilson et al.^29^ Indeed, creating molecules to have kinetic asymmetry is in fact the most difficult design task in making molecular motors This design principle for catalytically driven motors is in sharp contrast to the case of light-driven motors where a power stroke can be, and often is, a very important feature allowing directed motion^33^.

### The “local detailed balance” hypothesis is incorrect

The cycle from Fig. 3 can be written in linear form as

$$\rightleftarrows {\mathrm{p}}_{\mathrm{b}}\begin{array}{*{20}{c}} {K_{\mathrm{b}}} \\ \rightleftharpoons \\ {} \end{array}{\mathrm{d}}_{\mathrm{b}}\begin{array}{*{20}{c}} {\omega _{{\mathrm{d,}} + }} \\ \rightleftarrows \\ {\omega _{{\mathrm{d,}} - }} \end{array}{\mathrm{d}}_{\mathrm{f}}\begin{array}{*{20}{c}} {K_{\mathrm{f}}^{ - 1}} \\ \rightleftharpoons \\ {} \end{array}{\mathrm{p}}_{\mathrm{f}}\begin{array}{*{20}{c}} {\omega _{{\mathrm{p}}, + }} \\ \rightleftarrows \\ {\omega _{{\mathrm{p,}} - }} \end{array}{\mathrm{p}}_{\mathrm{b}} \rightleftharpoons$$

where the rate constants are $\omega _{{\mathrm{d,}} + } = (k_{{\mathrm{d,}} - 1} + k_{{\mathrm{d, + 2}}});\omega _{{\mathrm{d,}} - } = ([{\mathrm{S}}]k_{{\mathrm{d,}} + 1} + [{\mathrm{P}}]k_{{\mathrm{d,}} - 2});\omega _{{\mathrm{p,}} + } = ([{\mathrm{S}}]k_{{\mathrm{p,}} + 1} + [{\mathrm{P}}]k_{{\mathrm{p,}} - 2});\,{\mathrm{and}}\,\omega _{{\mathrm{p,}} - } = (k_{{\mathrm{p,}} - 1} + k_{{\mathrm{p, + 2}}})$ . As we saw in the section on the MM mechanism the ratios of these rate constants are given in terms of the kinetic asymmetry factors^15^ with $\frac{{\omega _{{\mathrm{d,}} + }}}{{\omega _{{\mathrm{d,}} - }}} = \left( {K_{{\mathrm{d,S}}}[{\mathrm{S}}]{\cal{A}}_{\mathrm{d}}} \right)^{ - 1}$ and $\frac{{\omega _{{\mathrm{p,}} + }}}{{\omega _{{\mathrm{p,}} - }}} = K_{{\mathrm{p,S}}}[{\mathrm{S}}]{\cal{A}}_{\mathrm{p}}$. There is a very commonly adopted, but incorrect, approximation known as the “local detailed balance”^31^ condition that asserts that the ratios of the $\omega$ must be such that $K_{\mathrm{b}}K_{\mathrm{f}}^{ - 1}\frac{{\omega _{{\mathrm{d,}} + }}}{{\omega _{{\mathrm{d,}} - }}}\,\frac{{\omega _{{\mathrm{p,}} + }}}{{\omega _{{\mathrm{p,}} - }}} = e^{{\mathrm{\Delta }}\mu }$, which is wrong. The “local detailed balance” relation can, however, hold approximately in certain limits. The correct ratio is $K_{\mathrm{b}}K_{\mathrm{f}}^{ - 1}\frac{{\omega _{{\mathrm{d,}} + }}}{{\omega _{{\mathrm{d,}} - }}}\,\frac{{\omega _{{\mathrm{p,}} + }}}{{\omega _{{\mathrm{p,}} - }}} = \frac{{{\cal{A}}_{\mathrm{p}}}}{{{\cal{A}}_{\mathrm{d}}}}$. For ${\mathrm{\Delta }}\mu > 0$, when $\frac{{k_{{\mathrm{p,}} - 1}}}{{k_{{\mathrm{p,}} + 2}}} \gg 1$ and $\frac{{k_{{\mathrm{d,}} - 1}}}{{k_{{\mathrm{d,}} + 2}}} \ll e^{ - {\mathrm{\Delta }}\mu }$, then $\frac{{{\cal{A}}_{\mathrm{p}}}}{{{\cal{A}}_{\mathrm{d}}}} \approx e^{{\mathrm{\Delta }}\mu }$. Unfortunately, making the local detailed balance approximation without explicitly mentioning the limits of validity, as is all too often done, obscures the importance of kinetic asymmetry. Some biomolecular motors, such as the FoF1 ATP synthase^34^, seem to operate in this thermodynamically controlled limit^13^ where forcing the motor mechanically backward results in synthesis^35^ of ATP as predicted by “local detailed balance”. Others, such as kinesin, do not^13^ as shown by the fact that backward motion is stimulated by ATP and is presumably accompanied by increasing rates of ATP hydrolysis, a behavior predicted by Astumian and Bier^15^, and observed experimentally by Carter and Cross^36^.

The coupling between ${\mathrm{\Delta }}\mu$ and directional motion enforced by “local detailed balance” is of the type described by Koenig^37^ as arising by a “stroke of the pen”, where the directionality is enforced at the whim of the theorist. When we consider instead the detailed kinetics based on the experimental example of Wilson et al.^29^ we find that with ${\mathrm{\Delta }}\mu > 0$, when $\frac{{k_{{\mathrm{d,}} - 1}}}{{k_{{\mathrm{d,}} + 2}}} \gg 1$ and $\frac{{k_{{\mathrm{p,}} - 1}}}{{k_{{\mathrm{p,}} + 2}}} \ll e^{ - {\mathrm{\Delta }}\mu }$, the ratio of kinetic asymmetry factors is well approximated as $\frac{{{\cal{A}}_{\mathrm{p}}}}{{{\cal{A}}_{\mathrm{d}}}} \approx e^{ - {\mathrm{\Delta }}\mu }$ predicting net flux to the left in the linear model. In other words, the direction of motion is controlled entirely by kinetic asymmetry. This fact is hidden by the common but ill-advised “local detailed balance” approximation in which it is assumed that the kinetic gating is perfect and drives motion in the direction desired by the theorist. The synthetic motor of Wilson et al.^29^ is far from the regime in which local detailed balance holds as a limit, as the gating is quite weak, and the rotor in fact undergoes counter-clockwise rotation—motion to the left in the linear model, opposite that predicted by naive adoption of “local detailed balance”. As is clarified by the correct expression for the directionality, a major goal for improving synthetic molecular machines is to design molecules to incorporate strong allosteric interactions by which to enhance kinetic gating.

### Kinetic lattice model and diffusion on an energy surface

**Fig. 4:**
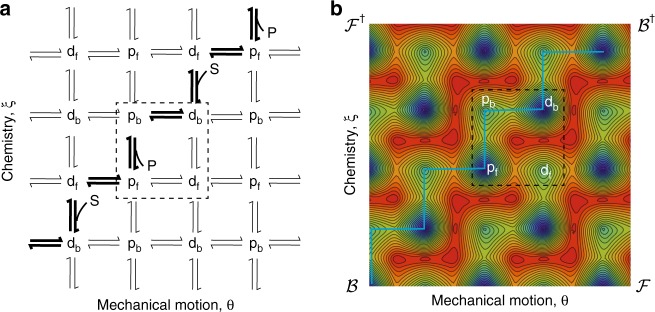
Kinetic lattice and energy landscape pictures showing how kinetic asymmetry leads to energy coupling. **a** A kinetic lattice model^15^ where the pathway favored by the kinetic asymmetry is shown in boldface. Having ${\mu }_{\mathrm{S}} \, > \, {\mu }_{\mathrm{P}}$ favors flux from top to bottom, and hence from right to left. **b** Contour plot of a 2-D energy landscape obtained by plotting the energies of the four states and six transition states and interpolating between the values^30^

A kinetic lattice model^15^ for the coupled processes of rotation (mechanical motion) and inter-conversion of S and P (chemistry) is shown in Fig. 4a. The pattern highlighted in boldface is selected by having the protecting group impede motion in one direction in the bound states, along with choosing $\left( {\frac{{k_{{\mathrm{d,}} - 1}}}{{k_{{\mathrm{d,}} + 2}}}} \right) > \left( {\frac{{k_{{\mathrm{p,}} - 1}}}{{k_{{\mathrm{p,}} + 2}}}} \right)$. This kinetically based selection has been accentuated relative to the rate constants in the experiment—the gating in the motor of Wilson et al.^29^ is actually quite weak. Although we speak of “cycles through the states”, there is no circulating flux in the kinetic lattice model for a catalysis-driven process. When $\mu _{\mathrm{S}} \, > \, \mu _{\mathrm{P}}$ there is a net tendency for downward flux, which in combination with the kinetic asymmetry causes the states to cycle predominately in the order ${{\mathrm{p}}_{\mathrm{f}} \to {\mathrm{d}}_{\mathrm{f}} \to {\mathrm{d}}_{\mathrm{b}} \to {\mathrm{p}}_{\mathrm{b}} \to {\mathrm{p}}_{\mathrm{f}}}$, while moving to the left, i.e., counter-clockwise rotation of the rotor. An energy landscape is obtained by assigning free-energies to the states consistent with the equilibrium constants ($K_{\mathrm{b}} = K_{\mathrm{f}}^{ - 1} = 100$) and to the transition states consistent with the asymmetry parameters $\frac{{k_{{\mathrm{p,}} + 2}}}{{k_{{\mathrm{p,}} - 1}}} = \frac{{k_{{\mathrm{d,}} - 1}}}{{k_{{\mathrm{d,}} + 2}}} = 100$ and to the barriers to rotation and then interpolating between these values as described in ref. ^30^. The landscape is neither an equilibrium nor a non-equilibrium model but rather a graphical display of the energies of the states and transition states. It is standard, but misguided, to incorporate the free-energy change Δ*μ* associated with the chemical reaction as a homogeneous force (net tilt) acting from top to bottom^13^ in the Fig. 4a. It is better to incorporate the modifications owing to having $\Delta \mu \ne 0$ on the probabilities in the theory ultimately based on this free-energy landscape. The structure (i.e., position of the catalytic site relative to the recognition sites) gives rise to the kinetically asymmetric characteristic of the energy landscape in Fig. 4b by which we recognize that the pathways ${\cal{B}}/{\cal{B}}^\dagger$ between the upper right and lower left corners are favored over the ${\cal{F}}/{\cal{F}}^\dagger$pathways between the upper left and lower right corners^32^. The zig-zag pattern is reminiscent of the energy landscapes of biomolecular machines, notably the F_o_F_1_ ATP synthase^34^. Having $\Delta \mu > 0$ then favors, by mass action (also known as Le Chatelier’s principle), the pathway ${\cal{B}}$, in which S is converted to P and the blue ring undergoes counter-clockwise revolution, over the pathway ${\cal{B}}^\dagger$ in which P is converted to S and the blue ring undergoes clockwise rotation.

## Catalysis-driven non-equilibrium adaptation

**Fig. 5:**
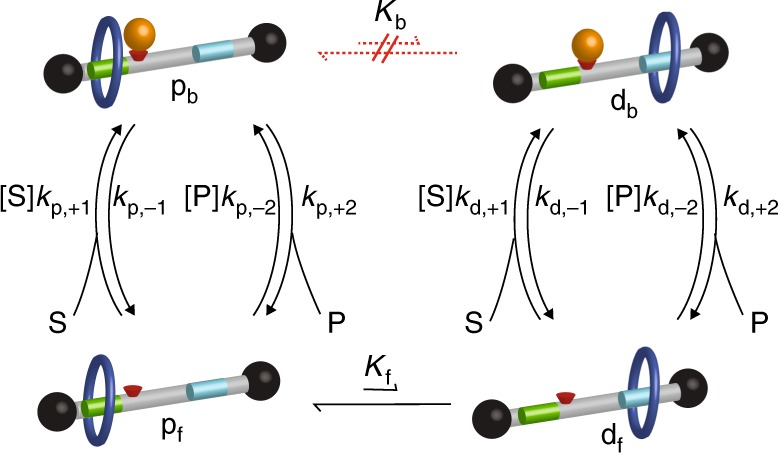
Catalysis-driven information ratchet can undergo non-equilibrium adaptation. Kinetic mechanism for transitions between the states of the molecule, where the rate constants for the catalytic reactions are shown explicitly. The direct transition between states p_b_ and d_b_ is blocked—both forward and backward rate constants are very small—but the equilibrium constant must nonetheless reflect the relative energies of the two states

In Fig. 3 the direct transitions between p_b_ and d_b_ are relatively fast, allowing the molecule to undergo directional cycling through the states where the condition of zero flux is $\frac{{{\cal{A}}_{\mathrm{p}}}}{{{\cal{A}}_{\mathrm{d}}}}e^w = 1$ and where each cycle can be used to do a maximum work $w_{\mathrm{max}} = ln\left( {\frac{{{\cal{A}}_{\mathrm{p}}}}{{{\cal{A}}_{\mathrm{d}}}}} \right)$ on the external environment. Similar ideas to those developed for the synthetic rotor can help provide understanding of non-equilibrium adaptation owing to catalysis of an exergonic chemical reaction. An example system is shown in Fig. 5, with the same driving reaction as in Fig. 3—the conversion of Fmoc-Cl to DBFV—and with the same recognition sites and asymmetrically located catalytic site but here incorporated in a rotaxane rather than a catenane. Although the key aspect of the molecular rotor described in Fig. 3 is unidirectional motion, the essence of thermodynamically non-equilibrium adaptation and self-assembly is the maintenance of a non-equilibrium distributions of state probabilities. As we will see, this disequilibrium can also be described in terms of the kinetic asymmetry parameters. A major difference between the catenane system of Fig. 3 and the rotaxane in Fig. 5 is that escapement directly between states ${\mathrm{p}}_{\mathrm{b}}$ and ${\mathrm{d}}_{\mathrm{b}}$ is so slow as to be virtually impossible. Consider the case that the blue ring is much more strongly attracted to the green recognition site than to the aqua recognition site such that both $K_{\mathrm{b}} \, < \, 1$ and $K_{\mathrm{f}} \, < \, 1$. At equilibrium we will have more molecules in the proximal than distal states $\left[ {{\mathrm{p}}_{\mathrm{b}}} \right] > \left[ {{\mathrm{d}}_{\mathrm{b}}} \right]$ and $\left[ {{\mathrm{p}}_{\mathrm{f}}} \right] > \left[ {{\mathrm{d}}_{\mathrm{f}}} \right]$. When ${\cal{A}}_{\mathrm{p}}{\cal{A}}_{\mathrm{d}}^{ - 1} \, < \, 1$ and away from equilibrium with $\Delta \mu > 0$, however, the kinetic asymmetry combines with catalysis tending to drive counter-clockwise rotation through the states ${{\mathrm{p}}_{\mathrm{b}} \to {\mathrm{p}}_{\mathrm{f}} \to {\mathrm{d}}_{\mathrm{f}} \to {\mathrm{d}}_{\mathrm{b}} \nrightarrow {\mathrm{p}}_{\mathrm{b}}}$. Because the transition ${{\mathrm{d}}_{\mathrm{b}} \rightleftharpoons {\mathrm{p}}_{\mathrm{b}}}$ is blocked in the rotaxane system in Fig. 5 the molecules tend to “pile up” in state ${\mathrm{d}}_{\mathrm{b}}$, forming a non-Boltzmann distribution. Using trajectory thermodynamics^38–40^ (see Box 2) the explicit expression for this non-equilibrium distribution is $\frac{{\left. {[{\mathrm{d}}_{\mathrm{b}}]} \right|_{{\mathrm{ss}}}}}{{\left. {[{\mathrm{p}}_{\mathrm{b}}]} \right|_{{\mathrm{ss}}}}} = K_{\mathrm{b}}{\cal{A}}_{\mathrm{d}}{\cal{A}}_{\mathrm{p}}^{ - 1}$, where we have taken the limit that the rate constants for the direct transition approach zero, preserving of course the ratio to be $K_{\mathrm{b}}$. For sufficiently large ${\cal{A}}_{\mathrm{d}}{\cal{A}}_{\mathrm{p}}^{ - 1}$ the ratio $\frac{{\left. {[{\mathrm{d}}_{\mathrm{b}}]} \right|_{{\mathrm{ss}}}}}{{\left. {[{\mathrm{p}}_{\mathrm{b}}]} \right|_{{\mathrm{ss}}}}}$ can be greater than unity despite the small equilibrium constant $K_{\mathrm{b}} < 1$. The system stores energy in the non-equilibrium distribution among the states^4^ given by $ln\left[ {\frac{{\left. {\left[ {{\mathrm{d}}_{\mathrm{b}}} \right]} \right|_{{\mathrm{ss}}}}}{{\left. {\left[ {{\mathrm{p}}_{\mathrm{b}}} \right]} \right|_{{\mathrm{ss}}}}}\frac{{\left. {\left[ {{\mathrm{p}}_{\mathrm{b}}} \right]} \right|_{{\mathrm{eq}}}}}{{\left. {\left[ {{\mathrm{d}}_{\mathrm{b}}} \right]} \right|_{{\mathrm{eq}}}}}} \right] = ln\left( {\frac{{{\cal{A}}_{\mathrm{d}}}}{{{\cal{A}}_{\mathrm{p}}}}} \right)$. The adaptive behavior is governed principally by kinetic asymmetry, not by the amount of energy absorption, and the system adjusts not to maximize dissipation of energy but to favor states with the greatest kinetic stability, i.e., with the longest lifetimes. With the direct transition ${{\mathrm{d}}_{\mathrm{b}} \rightleftharpoons {\mathrm{p}}_{\mathrm{b}}}$ kinetically blocked the lifetime of state ${{\mathrm{d}}_{\mathrm{b}}}$ is $\tau _{{\mathrm{d}}_{\mathrm{b}}} = \left( {k_{{\mathrm{d,}} - 1} + k_{{\mathrm{d,}} + 2}} \right)^{ - 1}$and that of state ${\mathrm{p}}_{\mathrm{b}}$ is $\tau _{{\mathrm{p}}_{\mathrm{b}}} = \left( {k_{{\mathrm{p,}} - 1} + k_{{\mathrm{p,}} + 2}} \right)^{ - 1}$. In the limit that the chemical potential difference between substrate and product is large, ${{\lim }}_{\Delta \mu \to \infty } \frac{{{\cal{A}}_{\mathrm{d}}}}{{{\cal{A}}_{\mathrm{p}}}} = \frac{{\tau _{{\mathrm{d}}_{\mathrm{b}}}}}{{\tau _{{\mathrm{p}}_{\mathrm{b}}}}}\frac{{k_{{\mathrm{p,}} - 1}}}{{k_{{\mathrm{d,}} - 1}}}$. Recently, Pascal and Pross^41,42^ have based an approach for describing the onset of complexity in terms of the amplification of kinetic asymmetry by replication, terming the adaptive behavior “dynamic kinetic stabilization” and offering insight into how simple matter can self-organize to become complex^43^ through catalysis with kinetic asymmetry.

The non-equilibrium distribution for the rotaxane in Fig. 5 persists only so long as Δ*μ* is maintained. If the chemical potential difference between substrate and product would relax to zero the state distribution would relax to equilibrium within several lifetimes $\tau _{\mathrm{d}_{\mathrm{b}}} + \tau _{\mathrm{p}_{\mathrm{b}}}$, generally a very short time, so storing molecules in strongly non-equilibrium states would seem to be impossible. Cheng et al.^44^ solved this problem brilliantly in constructing a synthetic molecular pump that uses chemical energy to pump a molecular structure into a strongly non-equilibrium state, combined with an ingenious incorporation of a steric speedbump to kinetically maintain the structure in a non-equilibrium form for months or years after the energy supply is turned off^44^. The approach of Cheng et al. used external manipulation of the redox potential of the system and is based not on an information ratchet but on an energy ratchet. The design, however, is amenable to being driven autonomously by catalysis, and comparison^17^ between the two approaches offers important insight into the essential feature of information ratchets by which all catalytically driven mechanisms for molecular machines must work.

Box 2 Trajectory thermodynamics and microscopic reversibility At thermodynamic equilibrium, the concentration distribution among different states of a macromolecule is determined by the free energies of the states and the chemical potentials of ligands without depending on detailed kinetics. A simple example is the elementary reaction ${\rm{p}}_{\rm{b}}\mathop { \rightleftharpoons }\limits^{K_{\rm{b}}} {\rm{d}}_{\rm{b}}$ in Fig. 5. When the chemical potentials of the ligands are chemo-stated to the same value, $\mu _{\rm{S}} = \mu _{\rm{P}} = \mu$, the concentration ratio between the two states is given by the equilibrium constant, $\frac{{\left. {[{\rm{d}}_{\rm{b}}]} \right|_{{\rm{eq}}}}}{{\left. {[{\rm{p}}_{\rm{b}}]} \right|_{{\rm{eq}}}}} = K_{\rm{b}} = e^{G_{{\rm{p}}_{\rm{b}}}^0 - G_{{\rm{d}}_{\rm{b}}}^0}$, irrespective of all other reactions in the system. As both p_b_ and d_b_ are bound states, this constant involves only the free energies of states p_b_ and d_b_, evaluated at the reference pressure and temperature.

Away from equilibrium, where $\mu _{\rm{S}} - \mu _{\rm{P}} = \Delta \mu \ne 0$, the simple expression for the ratio between the two states does not hold. Over the last several decades the concept of an equilibrium constant has been generalized to the possibly non-equilibrium steady state (ss) using the principle of microscopic reversibility^5,38^

8 $$\frac{{\left. {[{\rm{d}}_{\rm{b}}]} \right|_{{\rm{ss}}}}}{{\left. {[{\rm{p}}_{\rm{b}}]} \right|_{{\rm{ss}}}}} = K_{\rm{b}}\left \langle {e^{{\cal{W}}_{{\rm{neq}}}({\rm{p}}_{\rm{b}} \to {\rm{ d}}_{\rm{b}})}} \right \rangle_{\cal{S}}$$

The factor $\langle {e^{{\cal{W}}_{{\rm{neq}}}({\rm{p}}_{\rm{b}} \to {\rm{d}}_{\rm{b}})}}\rangle_{\cal{S}}$ is the exponential of the excess or non-equilibrium work exchanged with the environment averaged over all transition paths ${\cal{S}} ={ {\rm{p}}_{\rm{b}} \to \cdots \to {\rm{d}}_{\rm{b}}}$. Without repeating states there are five such paths or trajectories for the model in Fig. 5. Unlike *K*_b_, the factor $\langle {e^{{\cal{W}}_{{\rm{neq}}}({\rm{p}}_{\rm{b}} \to d_{\rm{b}})}}\rangle_{\cal{S}}$ is not a state function and depends on both the kinetics and the thermodynamics of the system. This factor is always unity at equilibrium, but away from equilibrium can be greater than, less than, or equal unity, depending on the kinetic asymmetry^15,16^ of the system. Even though in Fig. 1 states p_b_ and d_b_ are both bound and hence *K*_b_ is independent of the reference chemical potential *μ* of substrate, the ratio $\frac{{\left. {[{{\rm{d}}}_{\rm{b}}]} \right|_{{\rm{eq}}}}}{{\left. {[{{\rm{ p}}}_{\rm{b}}]} \right|_{{\rm{eq}}}}}$ depends on (adapts to) the chemical potential difference Δ*μ* through $\langle {e^{{\cal{W}}_{{\rm{neq}}}({\rm{p}}_{\rm{b}} \to {\rm{d}}_{\rm{b}})}}\rangle_{\cal{S}}$. The generalization of the equilibrium constant^38^ in Eq. 8 is based on the principle of microscopic reversibility according to which the probability, $\pi ({{\rm{p}}_{\rm{b}}\mathop { \to }\limits^{\cal{S}} {\rm{d}}_{\rm{b}}})$, of a specific trajectory, ${\cal{S}} \equiv{ {\rm{p}}_{\rm{b}} \to \cdots \to {\rm{d}}_{\rm{b}}}$, is related to the probability $\pi ({{\rm{d}}_{\rm{b}}\mathop { \to }\limits^{{\cal{S}}^\dagger } {\rm{p}}_{\rm{b}}})$

of the microscopic reverse (indicated by $\dagger$) of the trajectory, ${\cal{S}}^\dagger \equiv {\rm{d}}_{\rm{b}} \to \cdots \dagger \to {\rm{p}}_{\rm{b}}$ by the exponential of the free-energy exchanged between the macromolecule and its environment in the forward process^39^ ${\cal{W}}({{\rm{p}}_{\rm{b}}\mathop { \to }\limits^{\cal{S}} {\rm{d}}_{\rm{b}}})$,

9 $$\frac{{\pi \left( {{{\rm{p}}_{\rm{b}}\mathop { \to }\limits^{\cal{S}} {\rm{d}}_{\rm{b}}}} \right)}}{{\pi \left({{\rm{ {d}}_{\rm{b}}\mathop { \to }\limits^{{\cal{S}}^\dagger } p_{\rm{b}}}} \right)}} = e^{{\cal{W}}\left( {{{\rm{p}}_{\rm{b}}\mathop { \to }\limits^{\cal{S}} {\rm{d}}_{\rm{b}}}} \right)}$$

where the energy exchanged in the microscopic reverse trajectory is equal and opposite to that exchanged in the forward process, ${\cal{W}}({\rm{p}}_{\rm{b}}\mathop { \to }\limits^{\cal{S}} {{\rm{d}}}_{\rm{b}}) = - {\cal{W}}({{\rm{d}}_{\rm{b}}\mathop { \to }\limits^{{\cal{S}}^\dagger } {\rm{p}}_{\rm{b}}})$. We adopt a sign convention where ${\cal{W}}({\rm{p}}_{\rm{b}}\mathop { \to }\limits^{\cal{S}} {\rm{d}}_{\rm{b}})$ is positive when free-energy is transferred from the macromolecule to the environment in the trajectory ${\cal{S}}$. The probability $\pi \left( {{{\rm{p}}_{\rm{b}}\mathop { \to }\limits^{\cal{S}} {\rm{d}}_{\rm{b}}}} \right)$ is that for a specific trajectory ${\cal{S}}$, not the overall probability for state ${{\rm{p}}_{\rm{b}}}$ to undergo a transition to state ${{\rm{d}}_{\rm{b}}}$. We arrive at an expression of the second law by using these symmetries to write

10 $$\frac{{\pi \left( {{\rm{p}}_{\rm{b}}\mathop { \to }\limits^{\cal{S}} {\rm{d}}_{\rm{b}}} \right){\cal{W}}({\rm{p}}_{\rm{b}}\mathop { \to }\limits^{\cal{S}} {\rm{d}}_{\rm{b}}) + \pi \left( {{\rm{d}}_{\rm{b}}\mathop { \to }\limits^{{\cal{S}}^\dagger } {\rm{p}}_{\rm{b}}} \right){\cal{W}}({\rm{d}}_{\rm{b}}\mathop { \to }\limits^{{\cal{S}}^\dagger } {\rm{p}}_{\rm{b}})}}{{\pi \left( {{\rm{p}}_{\rm{b}}\mathop { \to }\limits^{\cal{S}} {\rm{d}}_{\rm{b}}} \right) + \pi \left( {{\rm{d}}_{\rm{b}}\mathop { \to }\limits^{{\cal{S}}^\dagger } {\rm{p}}_{\rm{b}}} \right)}} = \frac{{\left[ {1 - e^{ - {\cal{W}}({\rm{p}}_{\rm{b}}\mathop { \to }\limits^{\cal{S}} {\rm{d}}_{\rm{b}})}} \right]}}{{\left[ {1 + e^{ - {\cal{W}}({\rm{p}}_{\rm{b}}\mathop { \to }\limits^{\cal{S}} {\rm{d}}_{\rm{b}})}} \right]}}{\cal{W}}({\rm{p}}_{\rm{b}}\mathop { \to }\limits^{\cal{S}} {\rm{d}}_{\rm{b}}) \ge 0$$

for each trajectory ${\cal{S}}$, showing that the net flow of energy is, on average, always from the molecule to the environment until equilibrium is attained. The exchanged energy is the sum of a state function and a non-equilibrium work term^40^ ${\cal{W}}_{{\rm{neq}}} ({\rm{p}}_{\rm{b}}{\mathop { \to }\limits^{\cal{S}}} {\rm{d}}_{\rm{b}})$ that depends on the path ${\cal{S}}$,

11 $${\cal{W}}({\rm{p}}_{\rm{b}}\mathop { \to }\limits^{\cal{S}} {\rm{d}}_{\rm{b}}) = \underbrace {\left( {G_{{\rm{p}}_{\rm{b}}}^0 - G_{{\rm{d}}_{\rm{b}}}^0} \right)}_{{\rm{ln}}(K_{\rm{b}})} + {\cal{W}}_{{\rm{neq}}}\left( {{\rm{p}}_{\rm{b}}\mathop{\to}\limits^{\mathcal{S}}{\rm{d}}}_{\rm{b}} \right)$$

The steady-state ratio between states p_b_ and d_b_ can be calculated as the ratio of the sums of the probabilities of the trajectories from p_b_ to d_b_ and from d_b_ to p_b_,

12 $$\frac{{\left. {[{\rm{d}}_{\rm{b}}]} \right|_{{\rm{ss}}}}}{{\left. {[{\rm{p}}_{\rm{b}}]} \right|_{{\rm{ss}}}}} = \frac{{\mathop {\sum }\nolimits_{\cal{S}} \pi \left( {{\rm{p}}_{\rm{b}}\mathop { \to }\limits^{\cal{S}} {\rm{d}}_{\rm{b}}} \right)}}{{\mathop {\sum }\nolimits_{\cal{S}} \pi \left( {{\rm{d}}_{\rm{b}}\mathop { \to }\limits^{{\cal{S}}^\dagger } {\rm{p}}_{\rm{b}}} \right)}} = K_{\rm{b}}\underbrace {\mathop {\sum }\limits_{\cal{S}} \frac{{\pi \left( {{\rm{p}}_{\rm{b}}\mathop { \to }\limits^{\cal{S}} {\rm{d}}_{\rm{b}}} \right)e^{{\cal{W}}_{{\rm{neq}}}({\rm{p}}_{\rm{b}}\mathop { \to }\limits^{\cal{S}} {\rm{d}}_{\rm{b}})}}}{{\pi \left( {{\rm{p}}_{\rm{b}}\mathop { \to }\limits^{\cal{S}} {\rm{d}}_{\rm{b}}} \right)}}}_{\langle {e^{{\cal{W}}_{{\rm{neq}}}({\rm{p}}_{\rm{b}} \to {\rm{d}}_{\rm{b}})}}\rangle_{\cal{S}}}$$

recapitulating Eq. 8. The second equality arises by application of microscopic reversibility. The non-equilibrium term $\langle {e^{{\cal{W}}_{{\rm{neq}}}({{\rm{p}}_{\rm{b}}} \to d_{\rm{b}})}}\rangle_{\cal{S}}$ does not depend on the basic free-energies $G_{{\rm{p}}_{\rm{b}}}^0$ or $G_{{\rm{d}}_{\rm{b}}}^{0}$.

## Contrasting externally driven and catalysis-driven self-assembly

**Fig. 6:**
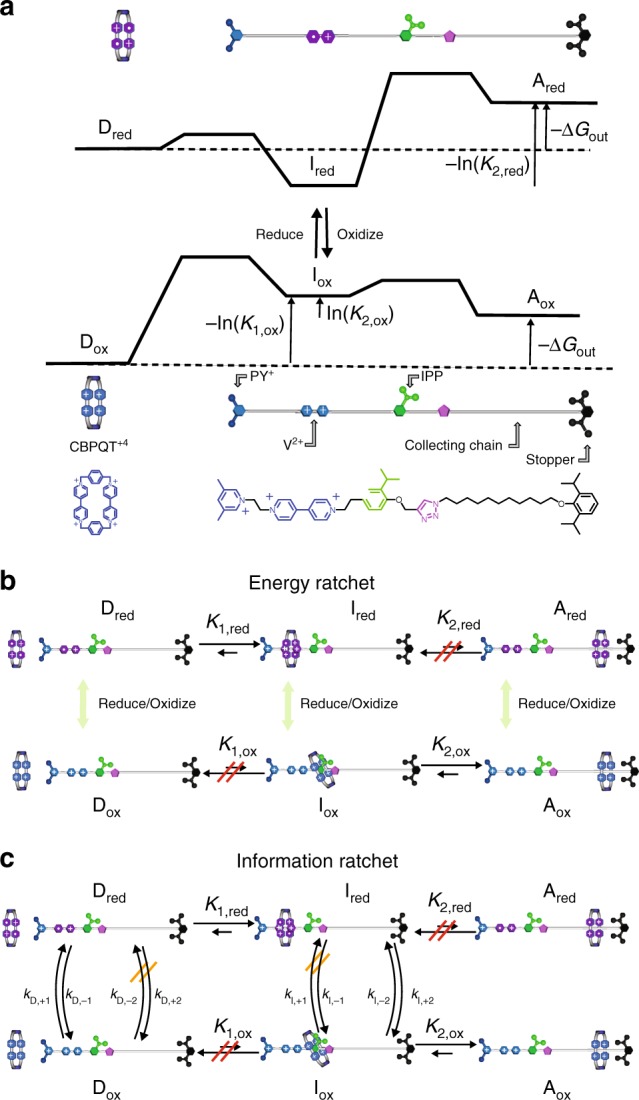
An artificial molecular pump driven by external modulation or catalyzed redox reaction. **a** Schematic depiction of the pump with energy profiles under oxidizing and reducing conditions, along with molecular structures. **b** Energy ratchet model for operation of the pump by externally changing the redox potential back and forth between reducing and oxidizing conditions, and **c** Information ratchet model^26^ for using energy from a redox reaction $${\mathrm{red}}_1 + {\mathrm{ox}}_2\begin{array}{*{20}{c}} {\Delta \mu } \\ \rightleftharpoons \\ {} \end{array}{\mathrm{red}}_2 + {\mathrm{ox}}_1$$ to drive assembly of CBPQT rings onto the collecting chain of the DB molecule, where disassembled (D), intermediate (I), and assembled (A) forms of the molecule when reduced (red) and when oxidized (ox) are shown

Both the molecular rotor (Fig. 3) and the adaptive switch (Fig. 5), like all catalysis-driven molecular machines, function as information ratchets where directionality, adaptation, or self-assembly is governed solely by kinetic asymmetry. In contrast, an externally driven molecular machine such as the synthetic pump-assembler^44^ designed and synthesized by Cheng et al.^44^, functions as an energy ratchet. The design considerations for energy ratchets are different than those for information ratchets. The pump-assembler of Cheng et al. is shown schematically and as chemical structures in Fig. 6a, where the device functions to use external energy to pump a cyclo-bisparaquat (CBPQT) ring from the bulk solution onto a collecting chain and maintain it there. The profiles describe the energy of a the CBPQT ring under either reducing (top) or oxidizing (bottom) conditions as it moves along the dumbbell (DB) rod. The molecule comprises a DB-shaped rod-like molecule with a pyridinium (Py^+^) pseudo-stopper (a group over which passage of the CBPQT is impeded but not totally precluded) on one end, followed by a viologen recognition site separated from a collecting chain by an isopropyl phenyl (IPP) molecular speedbump. The collecting chain is terminated by a stopper group over which the CBPQT cannot pass. When the moieties are reduced (indicated by purple color), the viologen and CBPQT strongly attract one another, forming a very stable interaction, and the barrier owing to the Py^+^ is relatively low. When oxidized, however, the interaction between CBPQT and V is destabilized—the CBPQT and viologen must part company—but the barrier owing to Py^+^ is very large so the CBPQT dissociates to the collecting chain. Without the IPP speedbump it would be possible to assemble the DB/CBPQT complex with one ring, but no more, where continued cycles of reduction and oxidation would see the CBPQT move back and forth between the V recognition site and the collecting chain. Cheng et al. incorporated the IPP as a barrier that interacts sterically with the CBPQT ring. This interaction is more or less independent of whether the CBPQT is oxidized or reduced so that relaxation of the ring from the collecting chain to the recognition site is very slow. This allows several (up to four so far) rings to be pumped onto the collecting chain and remain kinetically stable for a long time—months or even years—despite the fact that a configuration with even one ring on the collecting chain is thermodynamically unstable.

The synthetic pump functions as an energy ratchet in a straightforward way. When the system is reduced, a CBPQT ring is recruited to the V recognition site, most probably from the bulk. When the molecule is oxidized, the ring moves off the recognition site, and because of the Coulombic repulsion with the Py^+^ group it does so most probably onto the collecting chain. By cycling between strongly reducing and oxidizing conditions, a situation arises in which almost every DB molecule has at least one, and possibly several CBPQT rings on the collecting chain, even though at any fixed redox potential, almost none of the DB molecules would be assembled with a CBPQT ring on the collecting chain.

The two essential requirements for operation of this energy ratchet mechanism are the redox-dependent modulation of the relative barrier heights owing to Py^+^ and IPP, and the redox dependent modulation of the interaction energy between CBPQT and V. The correlation by which the Py^+^ barrier is relatively low when the CBPQT-V interaction is stable, and the IPP barrier is relatively low when the CBPQT-V interaction is unstable, is also key to the mechanism. The steady-state ratio between assembled and disassembled forms is approximately^5,45^ $\frac{{\left[ {\mathrm{A}} \right]|_{{\mathrm{ss}}}}}{{\left[ {\mathrm{D}} \right]|_{{\mathrm{ss}}}}} \approx K_{1,{\mathrm{red}}}K_{2,{\mathrm{ox}}}$, which is greater than unity even though the equilibrium constants under either reducing or oxidizing conditions are less than unity^45^ $\frac{{\left. {\left[ {\mathrm{A}} \right]} \right|_{{\mathrm{eq}}}}}{{\left. {\left[ \mathrm{D} \right]} \right|_{{\mathrm{eq}}}}} = K_{1,{\mathrm{red}}}K_{2,{\mathrm{red}}} = K_{1,{\mathrm{ox}}}K_{2,{\mathrm{ox}}} = e^{ - {\mathrm{\Delta }}G_{{\mathrm{out}}}} < < 1$.

It is tempting to extrapolate this energy ratchet picture to the design of an autonomous pump driven by catalysis of an exergonic redox reaction^45^, ${\mathrm{ox}}_1 + {\mathrm{red}}_2 \mathop{\rightleftharpoons}\limits^{\Delta \mu } {\mathrm{red}}_1 + {\mathrm{ox}}_2$, by the CBPQT ring. Indeed, with $\Delta \mu \gg 0$, the kinetic model in Fig. 6c predicts, with realistic and thermodynamically consistent parameters, almost certain formation of the assembled complex despite the large ${{\Delta }}G_{{\mathrm{out}}}$. However, when the kinetic equations are solved, the non-equilibrium steady-state ratio between assembled and disassembled components is seen to be determined solely by kinetic gating and is given by the expression^5,45,46^ $\frac{{\left. {\left[ {\mathrm{A}} \right]} \right|_{{\mathrm{ss}}}}}{{\left. {\left[{\mathrm{ D}} \right]} \right|_{{\mathrm{ss}}}}} \approx e^{ - {\mathrm{\Delta }}G_{{\mathrm{out}}}}\frac{{{\cal{A}}_{\mathrm{I}}}}{{{\cal{A}}_{\mathrm{D}}}}$, where $\left. {\left[ {\mathrm{A}} \right]} \right|_{{\mathrm{ss}}}\,{\mathrm{and}}\,\left. {\left[ {\mathrm{D}} \right]} \right|_{{\mathrm{ss}}}$ are the total concentrations of assembled and disassembled forms of the molecule, respectively, and where $\frac{{{\cal{A}}_{\mathrm{I}}}}{{{\cal{A}}_{\mathrm{D}}}}$ is independent of $K_{1,{\mathrm{red}}}K_{2,{\mathrm{ox}}}$. The Coulombic energy difference between the reduced and oxidized intermediate states has no role in determining the steady-state distribution, and the principle allowing a non-equilibrium assembled structure to undergo self-assembly is kinetic asymmetry by which the half-reaction ${\mathrm{red}}_1 \rightleftharpoons {\mathrm{ox}}_1$ occurs predominately in the disassembled (D) state, and the other half-reaction ${\mathrm{ox}}_2 \rightleftharpoons {\mathrm{red}}_2$ in the partly assembled (I) state. Attempts to design strong specificity via allosteric interactions remains a work in progress^47,48^. The states A_ox_ and A_red_ in Fig. 6c are excellent examples of kinetically stable but thermodynamically unstable states. In equilibrium at any redox potential there would be almost no molecules in the assembled states, but when powered with a large Δ*μ* the fraction of rotaxanes in states A_ox_ and A_red_ can be pumped to exceed 90% and most DB molecules will end up with several rings on the collecting chain. If the chemical fuel is removed the rotaxanes will remain indefinitely in state A because there is no kinetically accessible path for the ring to dissociate from the rod.

## A common misconception in dissipative self-assembly

**Fig. 7:**
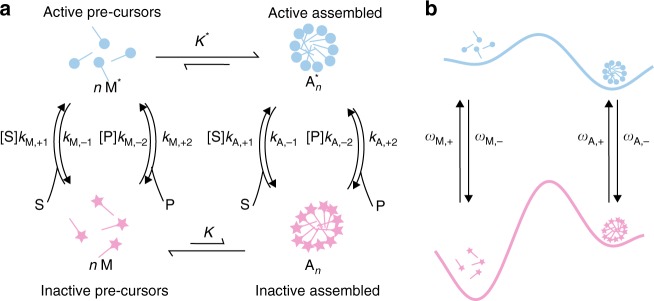
Typical description of dissipative self-assembly in which inactive precursors are activated to a configuration that spontaneously undergoes self-assembly. **a** Model showing the kinetic cycle by which assembly can be controlled. Especially if dissociation of inactive monomers (defects) from the assembled structure is slow, maintaining S and P out of equilibrium will result in assembly. **b** Energy landscape model illustrating the “just-so” story in which input energy is used to promote the inactive monomers to an active form that undergoes spontaneous (exergonic) transition to the deactivated form. In fact, when the energy is provided by catalysis of a chemical reaction the free-energy for assembly of the active monomers on the blue surface is absolutely irrelevant

Let us examine self-assembly driven by catalytic conversion of a chemical fuel such as ATP in a context often discussed in the recent literature. We imagine monomers “activated” by catalyzing a chemical reaction spontaneously undergoing self-assembly but eventually deactivating and disassembling spontaneously. Because of the spontaneous deactivation, continuous catalysis is needed to maintain the assembled structures, motivating the description “dissipative self-assembly”^49–51^ (DSA). Unfortunately, the heuristic paradigms that have emerged to describe so-called DSA can be misleading. In Fig. 7a, we see a kinetic scheme for dissipative self-assembly of phospholipids to form assembled vesicles. The basic idea is that “inactive” precursors (M) that have little tendency to form assembled structures ($K \ll 1$) are activated by catalysis of a chemical reaction, e.g. phosphorylation of the monomer when ATP is hydrolyzed to ADP to an active form M^*^. The activated monomers M^*^ have a strong affinity to form assembled structures ($K^* \gg 1$) and undergo spontaneous self-assembly to the *n*-mer ${\mathrm{A}}_n^ \ast$. In the absence of fuel, these assembled structures would eventually fall apart as the individual monomers undergo spontaneous deactivation (e.g., dephosphorylation in the case of the ATP hydrolysis driven system), so continuous catalysis and dissipation is necessary to maintain the non-equilibrium assembled state. A heuristic energy landscape is shown in Fig. 7b. The process appears to be diabatic, involving diffusion and thermal activation on two separate energy surfaces with some “energy input” mediating transition between the two surfaces, where the surface to surface transitions are parametrized by the transition constants $\omega _{{\mathrm{M, \pm }}}\,{\mathrm{and}}\,\omega _{{\mathrm{A, \pm }}}$. The intuitive understanding in which promotion to the “activated” (blue) surface facilitates spontaneous, energetically downhill assembly followed by deactivation that is motivated by this picture is correct for light-driven processes, where the *ω* are constrained by the Einstein relations for stimulation and emission of photons, and for externally driven processes, where the *ω* are governed by the settings on a robotic titrator that adds pulses of fuel^52^. The steady-state concentration ratio between assembled and disassembled forms in this case can be as large as K* if the disassembly A_*n*_→M is slow compared with other processes.

In contrast, when the energy input occurs through catalysis of a chemical reaction, irrespective of whether the reaction is ATP hydrolysis, conversion of Fmoc-Cl to DBFV, transport of proton across a membrane, or electron transfer, the seemingly common sense picture formed by considering the energy landscapes is wrong—the non-equilibrium behavior of the system is independent of the relative energies of the wells on the activated surface (shown in light blue in Fig. 7b), i.e., is independent of K*. This fact can be appreciated by application of microscopic reversibility Box 2. The key feature by which catalysis of S to P can drive assembly is kinetic asymmetry. If there is selective gating such that free monomers react rapidly with S but slowly with P, and assembled monomers react rapidly with P but slowly with S, then disequilibrium in the ${\mathrm{S}} \rightleftharpoons {\mathrm{P}}$ reaction drives formation of a non-equilibrium steady-state distribution. If assembly/disassembly of the inactive state is slow, catalysis will result in a steady-state distribution $K\frac{{{\cal{A}}_{\mathrm{M}}}}{{{\cal{A}}_{\mathrm{A}}}}$ that is multiplied by a factor $\frac{{{\cal{A}}_{\mathrm{M}}}}{{{\cal{A}}_{\mathrm{A}}}}$ relative to the equilibrium case, where ${\cal{A}}_{\mathrm{A}} = \frac{{\left( {\frac{{k_{{\mathrm{A}}, - 1}}}{{k_{{\mathrm{A}}, + 2}}} + e^{ - \Delta \mu }} \right)}}{{\left( {\frac{{k_{{\mathrm{A}}, - 1}}}{{k_{{\mathrm{A}}, + 2}}} + 1} \right)}}$ and ${\cal{A}}_{\mathrm{M}} = \frac{{\left( {\frac{{k_{{\mathrm{M}}, - 1}}}{{k_{{\mathrm{M}}, + 2}}} + e^{ - \Delta \mu }} \right)}}{{\left( {\frac{{k_{{\mathrm{M}}, - 1}}}{{k_{{\mathrm{M}}, + 2}}} + 1} \right)}}$. Despite appearances, the process is not diabatic (occurring on two separate 1-dimensional energy surfaces) but adiabatic (occurring on one two-dimensonal energy surface). The constraints of microscopic reversibility $K^{\ast} K^{ - 1}\frac{{\omega _{{\mathrm{A, + }}}\omega _{{\mathrm{M}}, - }}}{{\omega _{{\mathrm{M}}, + ,}\omega _{{\mathrm{A}}, - }}} = \frac{{{\cal{A}}_{\mathrm{M}}}}{{{\cal{A}}_{\mathrm{A}}}}$ on the transition constants assure that when the energy minima (states) and saddle points (transition states) are plotted then interpolation between these points results in an 2-D scalar energy surface for which the gradient of the energy is curl free.

## Conclusion

Thermodynamics—free-energy differences between states—dominates the properties of a macromolecular system at global equilibrium. Away from equilibrium, kinetic properties—the relative heights of energy barriers—come to center stage and determine not only the distribution among states, but also behavior such as directional motion^26^, adaptation^50^, self-assembly^4,51^. The take home message from this perspective article is encapsulated in Fig. 3. Let us consider the rotor at chemical equilibrium, where the chemical potential of Fmoc-Cl is equal the chemical potential of DBFV. Chemical equilibrium is dynamic, with every possible chemical and mechanical transition occurring, but where the probability for any transition is exactly the same as the probability for the microscopic reverse of that transition: sometimes, the blue ring moves clockwise from the green to the aqua site, and with equal probability moves counter-clockwise from the aqua to the green site; sometimes, an Fmoc binds at the active site when the blue ring occupies the green site, and with equal probability Fmoc dissociates from the active site when the blue ring occupies the green site; sometimes, when the active site is free the blue ring moves clockwise from the aqua site to the green site, and with equal likelihood the blue ring moves counter-clockwise from the green site to the aqua site when the active site is free etc. At thermodynamic equilibrium each of the six forward transitions in Fig. 3a is exactly as likely as its microscopic reverse. As a result of this global detailed balance no net progress is made by the rotor in one direction or the other. What happens when Fmoc, but not DBFV, is added and chemo-stated to a new higher level to maintain the system away from chemical equilibrium. Does the character of the mechanical transitions change? Of course not. Does Fmoc violently kick a blue ring at the green site when it binds to the active site? Absurd! The only thing that changes is that the probability to bind Fmoc is increased, and therefore the probabilities of all trajectories involving binding Fmoc and release of DBFV are increased relative to their microscopic reverses in which DBFV binds and Fmoc is released.

The kinetic rate constants in Fig. 3, along with constraints Eq. (5) and (6) reflect quantitatively this verbally described thermodynamic reality. The direction of rotation of the dark blue ring owing to catalysis of Fmoc to DBFV depends only on whether the ratio of the “off ” rate constants, $\frac{{k_{{\mathrm{p,}} + 2}k_{{\mathrm{d,}} - 1}}}{{k_{{\mathrm{p,}} - 1}k_{{\mathrm{d,}} + 2}}}$, is less than or greater than unity. The direction of rotation does not depend on the free-energies of the states or on either $K_{\mathrm{b}}\,{\mathrm{or}}\,K_{\mathrm{f}}$. The mechanism by which rotation occurs is an information ratchet^6,17,18,21,23,52^, where the “information” is the dependence of the rate of binding Fmoc on the location of the ring, and the net rotation is owing to biased diffusion of the blue ring relative to the larger ring. The conclusion that directed motion can only occur by an information ratchet mechanism holds for all molecular machines and systems that are driven by catalysis of a chemical reaction.

A key goal for chemists will be to explore approaches by which allosteric interactions can be engineered in synthetic systems^48^. Even systems as complicated as DNA unwinding helicases^53^ and the ribosome^54,55^ operate as Brownian information ratchets, and in recent work, Goloubinoff et al.^56^ have shown that Chaperones can use an information ratchet mechanism to allow ATP hydrolysis to stabilize proteins in a non-equilibrium distribution of structures even under denaturing conditions. Catalysis of an exergonic reaction allows proteins not only to walk in a hurricane^26^, but also to fold in a stiff wind^57^.

Unfortunately, if one chooses to incant the words “far from equilibrium” as an excuse to assign plausible but wrong “common sense” rate constants for models similar to that in Fig. 3 rather than rate constants that are thermodynamically consistent, it is easy to arrive at the erroneous conclusion that the directionality is opposite to that observed and is governed by the free-energies of the states (i.e., the product of the equilibrium constants for the conformational changes, $K_{\mathrm{b}}K_{\mathrm{f}}^{ - 1}$). This false conclusion is seemingly supported by systems with similar kinetic mechanisms, but which are driven by external modulation or by light and where the directionality is, in fact, governed by $K_{\mathrm{b}}K_{\mathrm{f}}^{ - 1}$. For light or externally driven systems, the mechanism by which directional motion occurs is best described as an energy ratchet^2,17,45^. The rate constants for the pumping transitions are governed by different constraints than microscopic reversibility—the Einstein relations for light-driven processes, or the knob on the robotic titrator in the case of external modulation. The constraints on the rate constants make all the difference in the world with regard to interpreting the behavior of molecular machines. This was re-emphasized in Fig. 6b, c, where the electrostatic repulsion between the CBPQT ring and the viologen recognition site when both are oxidized was shown to be essential for externally driven pumping, but plays no role in determining the steady-state distributions in the context of an, as yet hypothetical, catalysis-driven mechanism.

When framed in the context of a graphical description shown in Fig. 7 such energy ratchets can lead to seductively plausible verbal stories of how a chemical catalysis-driven mechanism might operate. Unfortunately, such narratives give substance to the graphical description shown in Fig. 7, which can be a “trompe l’oeil*”* that fools the eye into believing an analogy between light and externally driven molecular machines on the one hand and catalysis-driven molecular machines on the other hand. As University of Chicago historian and Librarian of Congress (1975–1987) Daniel Boorstin saliently observed, “the greatest enemy of knowledge is not ignorance, it is the illusion of knowledge.” Depictions similar to Fig. 7 have been given for many, if not all, biomolecular machines—muscle, kinesin, the flagellar motor, FoF1 ATPase, etc. A recent author has even described the phosphorylated form of the FoF1 ATPase as being an “excited” state^58^. This is an abuse of terminology that is profoundly wrong and misleading.

The best protection against incorrect kinetic modeling is strict enforcement of the principle of microscopic reversibility^6,16,59,60^ as discussed in Box 2. Microscopic reversibility provides a solid foundation for the framework of a comprehensive kinetic and thermodynamic description of molecular machines^2,13,16,32^. It is also essential to incorporate the molecular details of binding/dissociation of substrate and dissociation/binding of product in the mechanism before making approximations and taking limits, which must be done in a systematic and principled way. This framework is key to interpretation of the results of computational studies of the energy landscapes of biological molecular machines^61–64^, where recent work shows that many, if not all, enzymes undergo directional motion during catalysis^65^. A major insight provided by computational studies of catalytic biomolecular machines^61–64^ is that the conformational motion can be described as diffusion on a single multidimensional energy surface *U*(*r*), and that the velocity *dr*/*dt* is proportional to the gradient of the potential^9^ $\nabla U(r)$. This proportionality, combined with the vector identity $\nabla \times \nabla U\left( r \right) = 0$, is at the heart of many of the corollaries of microscopic reversibility on which we rely in the development of the general theory for processes driven by disequilibrium of the reaction that the machine catalyzes^13^.

Notably, the response (adaptation) of a catalytic macromolecule to Δ*μ* is independent of the free-energies of the states and depends only on the relative transition state energies. We can understand this by recognizing that a state is a region on an energy surface where the local energy is a minimum, and where the energy increases in every direction, i.e., the energy is locally symmetric. In contrast a transition state is a saddle point at which the energy decreases in either direction along one coordinate and increases in both directions along other orthogonal coordinates, thus imbuing the transition state with an intrinsic directional asymmetry^30^. It is this intrinsic asymmetry that is exploited by information ratchets^17,32^ to kinetically select one path over other, possibly thermodynamically equivalent or even preferred, paths where the selection between paths is based on which has the lowest energy barrier^62^.

The equilibrium character of the mechanical motions by which molecular machines carry out their function requires a very different set of concepts for analysis of the thermodynamic performance of non-equilibrium molecular systems than for macroscopic devices. For macroscopic machines the minimization of friction is key to optimizing the efficiency. On the other hand, friction has little or no role beyond setting the timescale of the rate constants for molecular machines. The protein motions are exactly the same at and away from thermodynamic equilibrium—only the relative probabilities of those motions are changed when the system is away from thermodynamic equilibrium—and the mechanism by which kinetic gating works is well described as the biased diffusion of parts of the protein relative to other parts on an energy landscape. No fundamental limit other than unity can be expected for the thermodynamic efficiency [for very strong kinetic asymmetry the efficiency is $\eta = \frac{w}{{\Delta \mu }}\tanh \left( {\frac{{\Delta \mu - w}}{2}} \right)$, which for general Δ*μ* and *w* can approach unity but for $\Delta \mu = 20$ (the energy released by ATP hydrolysis) is maximized at 0.78 with $w = 16.5$, and the most appropriate parametrization of the effectiveness of energy transduction is the ratio of the minimum amount of energy necessary to accomplish a specific task divided by the actual energy used—the generalized efficiency^18^. The major mechanism for energy loss is slip—undergoing cycles in which chemical fuel is dissipated but no useful work is accomplished by the macromolecular system. The key principle necessary for kinetic gating and for the use of “information” in macromolecules is well established to be allosteric interaction^26,48^ by which the occupancy at one site influences lability and stability at a remote site and vice versa.

The study of thermodynamically non-equilibrium molecular systems has been greatly stimulated by the recent award of the Nobel prize to Jean Pierre Sauvage^66^, Ben Feringa^67^, and Fraser Stoddart^68^ for design and synthesis of molecular machines, many of which take inspiration from biology^69^. In particular, the side by side comparison^70^ of a light-driven motor and a chemical catalysis-driven motor^29^ clarifies that the “power-stroke” idea that has dominated mechanistic discussions of molecular motors such as the F_o_F_1_ ATPase, actin-myosin, kinesin, the flagellar motor, and other biomolecular motors since the 1950’s is simply wrong^13,30,46^. These catalysis-driven motors work instead by kinetic gating. The selection mechanism between those trajectories in which ATP is hydrolyzed and those trajectories in which ATP is synthesized is known as mass action (Le Chatlier’s principle). The selection between those trajectories in which ATP is hydrolyzed and the molecule moves forward or rotates clockwise and those trajectories in which ATP is hydrolyzed and the molecule moves backward or rotates counter-clockwise is achieved by kinetic asymmetry in which one of the trajectories has a lower activation barrier than the other^13,30^. There is not, nor can there be, a selection for catalysis-driven processes based on a power-stroke or any other principal that relies on differences between the free energies of states^30^.

Although thermodynamic equilibrium is unique, there are many types of disequilibria, and the lessons learned from one type, e.g., from light-driven processes, or from externally driven motors and pumps^13,16^, do not in general transfer to other types, e.g., to processes driven by catalysis of a chemical reaction^16^. Further progress in understanding will doubtless result from ongoing advances in design and experimental synthesis of different types of molecular machines^17,19,67,68^, non-equilibrium self-assembling structures^4,50,51^, and fuel driven adaptive systems^71–73^. It is perhaps not overly optimistic to imagine that soon it will be possible to catch a glimpse, if only on the distant horizon, of an answer to the question of how simple matter becomes complex^43^. This will likely involve a replication mechanism^74,75^, and insight into the effects of kinetic asymmetry^15,16^, allostery^76,77^, escapement and disequilibria^78,79^, and dynamic kinetic stabilization^41,42^ will have a critical role in designing such systems.
